# Anatomy, Imaging, and Clinical Significance of the Cervicothoracic (Stellate) Ganglion

**DOI:** 10.3390/diagnostics15222911

**Published:** 2025-11-17

**Authors:** Mugurel Constantin Rusu, Ionuţ Mădălin Munteanu, Alexandra Diana Vrapciu, Adelina Maria Jianu, Sorin Hostiuc, Răzvan Costin Tudose, Andrei Gheorghe Marius Motoc

**Affiliations:** 1 Division of Anatomy, Department 1, Faculty of Dentistry, “Carol Davila” University of Medicine and Pharmacy, 050474 Bucharest, Romania; mugurel.rusu@umfcd.ro (M.C.R.); razvan-costin.tudose0721@stud.umfcd.ro (R.C.T.); 2 Institute for Cardiovascular Diseases of Timişoara, Clinic of Anesthesia and Intensive Care, “Victor Babes” University of Medicine and Pharmacy Timişoara, Gheorghe Adam Street, No. 13A, 300310 Timişoara, Romania; 3 Department of Anatomy and Embryology, Faculty of Medicine, “Victor Babeș” University of Medicine and Pharmacy, 300041 Timișoara, Romania; 4 Division of Legal Medicine and Bioethics, Faculty of Dentistry, “Carol Davila” University of Medicine and Pharmacy, 050474 Bucharest, Romania; sorin.hostiuc@umfcd.ro; 5 Research Department, “Dr. Carol Davila” Central Military Emergency Hospital, 010825 Bucharest, Romania

**Keywords:** sympathetic trunk, vertebral artery, cardiac nerves, vertebral nerve, subclavian ansa, stellectomy, stellate ganglion block

## Abstract

**Background/Objectives**: The stellate ganglion (SG), formed by the fusion of the inferior cervical and first thoracic sympathetic ganglia in approximately 80% of individuals, plays crucial roles in cardiac innervation, pain management, and autonomic regulation. This review examines the anatomical variations, histological structure, clinical applications, and therapeutic implications of the SG and stellate ganglion block (SGB), presenting original high-resolution magnetic resonance imaging (MRI) evidence of SG visualization, an underutilized approach in autonomic nervous system research. **Methods**: We conducted a comprehensive literature review of anatomical, physiological, and clinical studies on the SG, incorporating original anatomical dissections and high-resolution MRI. Contemporary research on SGB applications, complications, and mechanisms of action was analysed and correlated with imaging characteristics. **Results**: The SG demonstrates significant anatomical variability, including the presence of intermediate ganglia, accessory nerve pathways, and variable relationships with surrounding vascular structures. Our original MRI imaging consistently identified the SG at the thoracic inlet, anterior to the neck of the first rib, lateral to the longus colli muscle, and posterior to the vertebral artery, demonstrating that advanced imaging can reliably visualize this critical autonomic structure and its anatomical variants. Histologically, it contains typical sympathetic architecture, comprising postganglionic neurons, satellite glial cells, and specialized SIF cells that modulate ganglionic transmission. SGB shows therapeutic efficacy across diverse conditions, including cardiac arrhythmias, chronic pain syndromes, post-traumatic stress disorder, sleep disorders, and immune dysfunction. The procedure’s mechanisms involve both direct sympathetic blockade and complex neuroimmune pathways that affect central autonomic centers and lymphoid organs. Complications include vascular injury, pneumothorax, and nerve blocks affecting the recurrent laryngeal and phrenic nerves. **Conclusions**: The SG represents a critical autonomic structure with expanding clinical applications. This work advances the field by demonstrating that high-resolution MRI can consistently and non-invasively visualize the SG and its anatomical variations, knowledge previously mostly limited to cadaveric studies. Understanding these imaging-defined anatomical variations is essential for optimizing therapeutic interventions. Advanced imaging guidance integrated with comprehensive anatomical knowledge is crucial for maximizing efficacy while minimizing complications in stellate ganglion block procedures.

## 1. Introduction

The cervicothoracic (stellate) ganglion (SG) is formed by the fusion of the inferior cervical and first thoracic sympathetic ganglia in approximately 80% of individuals [1]. Despite its clinical importance, significant anatomical controversies persist regarding its formation, relationships, and variations [2,3,4].

The SG gained increased clinical attention due to expanding applications of the stellate ganglion block (SGB) beyond traditional pain management [5,6,7]. Recent studies demonstrated efficacy in cardiac arrhythmias [8,9,10], sleep disorders [11,12,13,14,15,16], and other conditions [5,17]. However, anatomical variability has a direct impact on procedural success [3,18,19].

Key anatomical controversies include (1) the prevalence of intermediate ganglia [4,20,21], (2) the clinical implications of the nerve of Kuntz [19,22,23,24,25,26], (3) vertebral artery variations [27,28,29,30,31,32,33,34], and (4) optimal imaging approaches [35,36,37,38].

Objectives: This review aims to (1) systematically describe the SG anatomy based on contemporary cadaveric and imaging studies, (2) clarify anatomical variations using original dissections and literature synthesis, (3) correlate anatomical findings with imaging characteristics [36,37], (4) analyse clinical implications for interventional procedures [5,18], and (5) identify knowledge gaps.

## 2. Methods

A comprehensive literature search was conducted using PubMed/MEDLINE, Scopus, Web of Science, and Google Scholar databases from inception through June 2025. Search terms included combinations of “stellate ganglion,” “cervicothoracic ganglion,” “inferior cervical ganglion,” “sympathetic trunk,” “stellate ganglion block,” “cardiac sympathetic innervation,” “nerve of Kuntz,” “intermediate ganglion,” and “vertebral nerve.”

Inclusion criteria: (1) peer-reviewed articles in English; (2) anatomical studies using human cadavers or clinical imaging; (3) clinical studies on SGB applications; (4) physiological and experimental studies on SG function. Exclusion criteria: (1) non-English publications without available translations; (2) abstracts without full text; (3) case reports with fewer than 3 patients (unless reporting unique anatomical variants or rare complications).

Original dissection studies were conducted by the authors on formalin-fixed human cadavers. MRI imaging was obtained from institutional archives with appropriate consent. Historical anatomical texts and illustrations were used to provide context for the evolving understanding of SG anatomy.

## 3. Anatomical Considerations

### 3.1. Anatomical Terminology

The Terminologia Anatomica lists just the following terms related with the cervical sympathetic trunk (Latin Term/Latin Synonym/English Term): ganglion cervicale superius/-/superior cervical ganglion; ganglion cervicale medium/-/middle cervical ganglion; (ganglion vertebrale)/-/(vertebral ganglion); (ganglion cervicale inferius)/-/(inferior cervical ganglion); ganglion cervicothoracicum/ganglion stellatum/cervicothoracic ganglion; nervus jugularis/-/jugular nerve; nervus caroticus internus/-/internal carotid nerve; nervi carotici externi/-/external carotid nerves; nervi laryngopharyngei/rami laryngopharyngei/laryngopharyngeal nerves; nervus cardiacus cervicalis superior/-/superior cardiac cervical nerve; nervus cardiacus cervicalis medius/-/middle cervical cardiac nerve; ansa subclavia/-/ansa subclavia/Ansa Vieussenii/Ansa of Vieussens; nervus cardiacus cervicalis inferior/-/inferior cardiac cervical nerve; nervus vertebralis/-/vertebral nerve [39].

### 3.2. Basic Anatomy

The cervical portion of the sympathetic trunk (Figure S1) is characterized by the absence of segmental ganglia and of white communicating branches [40]. It is typically described as presenting three ganglia: the superior cervical ganglion, the middle cervical ganglion, and the cervicothoracic (stellate) ganglion (SG) [1]. The SG results from the fusion of the inferior cervical ganglion with the 1st or, in many cases, 2nd thoracic ganglion [1].

Kiray et al. (2005) found three sympathetic ganglia (superior, middle, and SG) in just 20.8% of specimens [41]. The sympathetic cervical trunk had just two ganglia, superior and SG, in 45.8% of specimens [41]. Superior, middle, vertebral, and SG were found in 12.5% of specimens, and superior, vertebral, and SG in 20.8% of specimens [41]. In Kawashima’s anatomical study (2005), the superior cervical ganglion was present bilaterally in all 18 embalmed adult human cadavers examined under a stereomicroscope [42]. The middle cervical ganglion was identified in 91.7% of sides (33/36), while a vertebral ganglion was found in 94.4% (34/36). Accessory structures include an additional middle cervical ganglion in 30.6% of cases (11/36) and a combined middle cervical-vertebral ganglion in 5.6% (2/36) [42].

**Figure 1:**
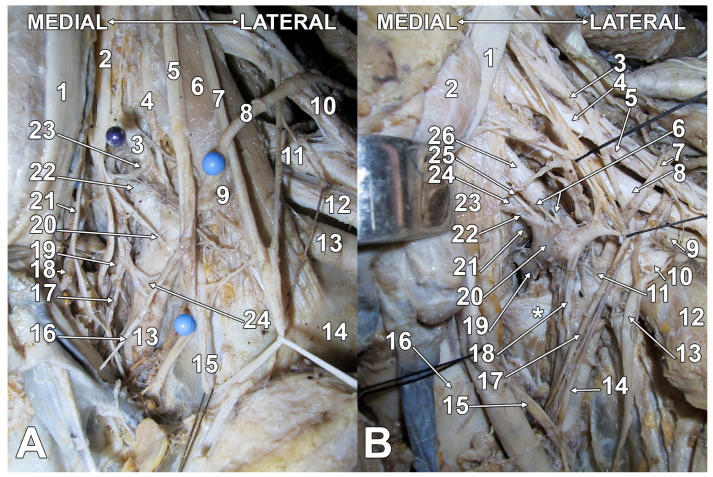
Original staged dissection of the left sympathetic trunk and stellate ganglion. Anterior views. (**A**). The scalenovertebral triangle. 1. common carotid artery; 2. cervical sympathetic trunk; 3. inferior thyroid artery; 4. ascending cervical artery; 5. vagus nerve; 6. anterior scalene muscle; 7. phrenic nerve; 8. transverse cervical artery; 9. suprascapular artery (cut); 10. superior trunk of the brachial plexus; 11. middle trunk of the brachial plexus; 12. inferior trunk of the brachial plexus; 13. subclavian artery; 14. first rib; 15. internal thoracic artery and plexus; 16. inferior vagal cervical cardiac branch; 17. communicating branch of the stellate ganglion with the superior cervical cardiac nerve; 18. thoracic duct; 19. upper pole of the stellate ganglion; 20. subclavian ansa; 21. superior cardiac cervical nerve; 22. vertebral artery; 23. inferior thyroid artery plexus; 24. subclavian artery plexus. (**B**). The supra-retro-pleural fossa (Sebileau-Ionescu). 1. common carotid artery; 2. thyroid lobe; 3. C5 nerve; 4. C6 nerve; 5. superior trunk of the brachial plexus; 6. vertebral artery plexus; 7. transverse cervical artery; 8. middle trunk of the brachial plexus; 9. inferior trunk of the brachial plexus; 10. subclavian artery; 11. subclavian artery plexus; 12. anterior scalene muscle (reflected antero-inferiorly); 13. phrenic nerve; 14. superior cervical cardiac nerve; 15. vagus nerve; 16. trachea; 17. middle cardiac cervical nerve; 18. inferior cardiac cervical nerve; 19. T1 sympathetic ganglion; 20. inferior cervical ganglion; 21. posterior end of the first rib; 22. communicating ramus with the C8 nerve; 23. longus colli muscle; 24. C8 nerve; 25. vertebral nerve; 26. vertebral artery. In (**B**) is indicated the suprapleural membrane (*).

**Figure 2:**
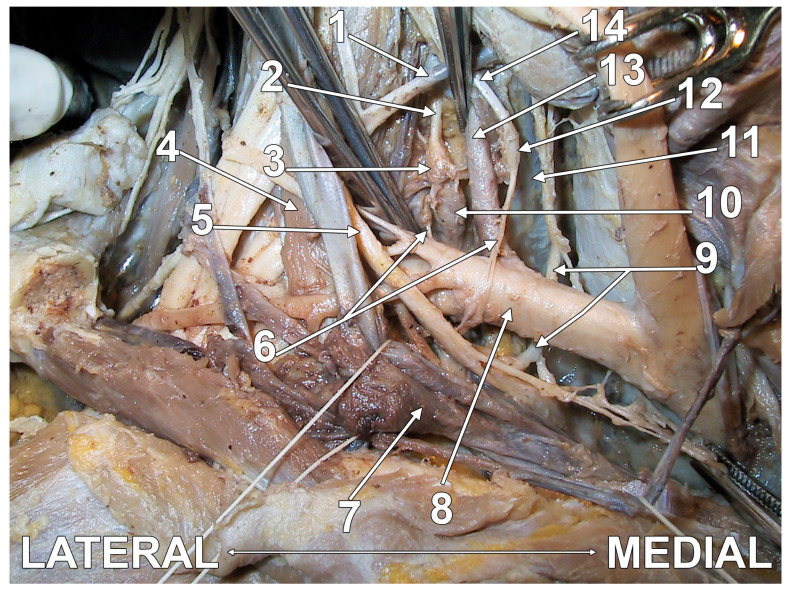
Original dissection of the inferior cervical and superior thoracic ganglia—incomplete stellate ganglion formation, and ansa subclavia. Anterior view. Right side. 1. inferior thyroid artery; 2. vertebral nerve; 3. inferior cervical ganglion; 4. anterior scalene muscle; 5. vagus nerve; 6. ansa subclavia; 7. brachiocephalic (innominate) vein; 8. subclavian artery; 9. recurrent laryngeal nerve; 10. T1 sympathetic ganglion; 11. longus colli muscle; 12. intermediate (vertebral) ganglion; 13. vertebral artery; 14. cervical sympathetic trunk.

**Figure 3:**
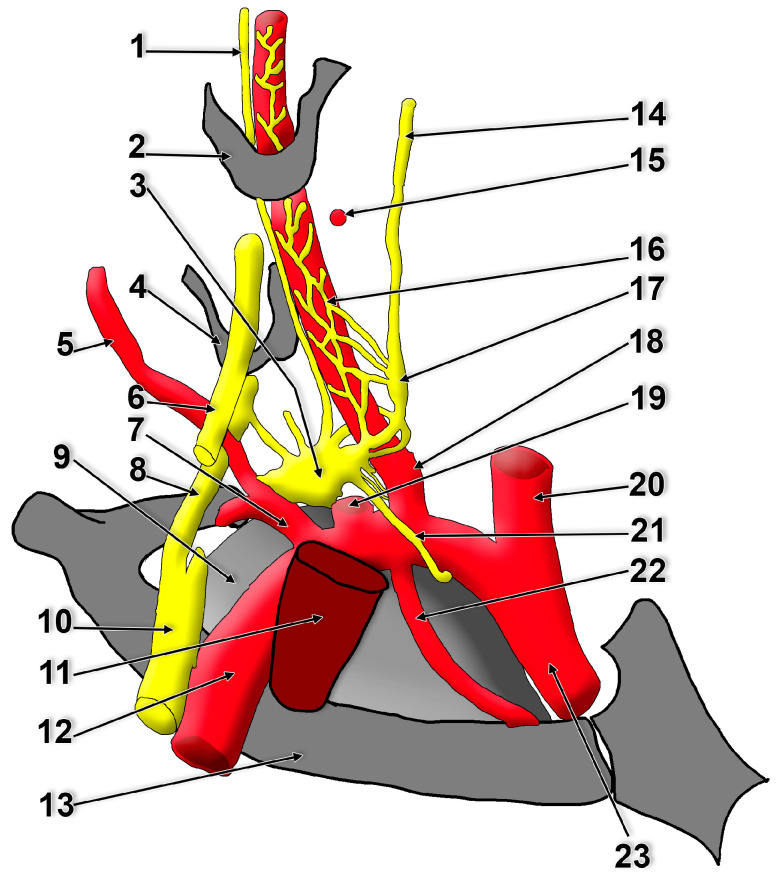
Lateral view of the right supra-retro-pleural fossa. Modified after Paturet (1964) [57]. 1. vertebral nerve; 2. C6 transverse process; 3. stellate ganglion; 4. C7 transverse process; 5. deep cervical artery; 6. C7 spinal nerve; 7. costocervical trunk; 8. C8 spinal nerve; 9. pleural dome; 10. inferior trunk of the brachial plexus; 11. anterior scalene muscle; 12. subclavian artery; 13. first rib; 14. cervical sympathetic trunk; 15. inferior thyroid artery; 16. vertebral artery plexus; 17. vertebral ganglion; 18. vertebral artery; 19. thyrocervical trunk; 20. common carotid artery; 21. subclavian ansa; 22. internal thoracic artery; 23. brachiocephalic trunk.

**Figure 4:**
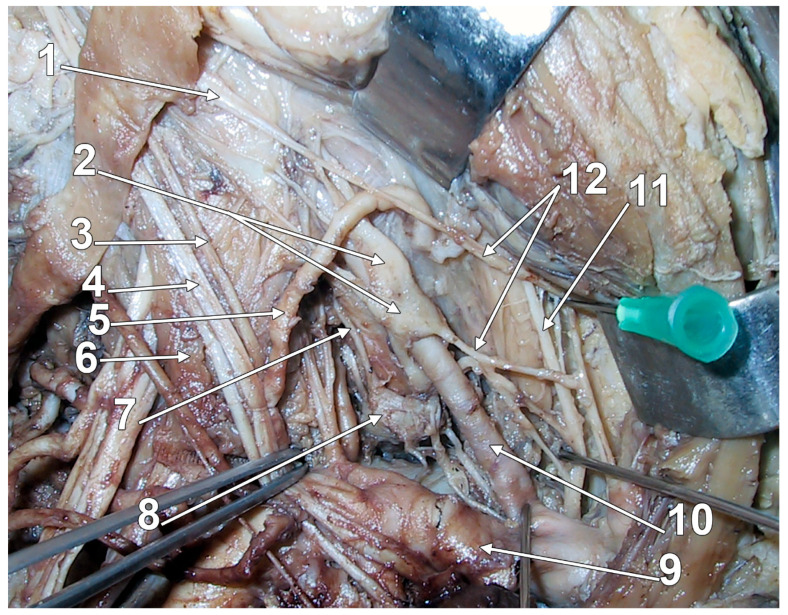
Original dissection of the sympathetic ganglia in the right scalenovertebral triangle. Right side. Anterior view. Original evidence. 1. sympathetic trunk; 2. fused middle cervical and vertebral ganglia; 3. ascending cervical artery; 4. vagus nerve; 5. inferior thyroid artery; 6. anterior scalene muscle; 7. vertebral nerve; 8. inferior cervical ganglion; 9. subclavian artery; 10. vertebral artery; 11. recurrent laryngeal nerve; 12. cervical cardiac nerves.

The morphology and topography of the SG are variable (Table 1, Figure 1, Figure 2, Figure 3 and Figure 4). The SG is typically located anterior to the neck of the first rib [3]. Some studies report the SG is more frequently found on the left side, especially in females, and is generally larger in males [23]. The SG is therefore situated at the C7-T1 vertebral junction, but with positional variations depending on the composition of the thoracic ganglia [42]. It is thus a bilateral structure. The SG results from the eventual fusion (80%) of the inferior cervical ganglion with the first, or in approximately 75% of cases, the first and second thoracic sympathetic ganglia [1,36,43,44]. Sometimes it may include the third and fourth thoracic ganglia [45]. Clinically, the term “SG” is sometimes used to indicate the inferior cervical and first thoracic ganglia, whether or not they are fused [46]. A constriction on the SG usually marks the borderline between the upper, cervical part of the SG and the lower, thoracic part of it [4].

An isolated inferior cervical ganglion without thoracic fusion was documented by Kawashima (2005) in 13.9% of specimens (5/36), while the majority demonstrated SG formation in 86.1% (31/36) [42]. Among the fused ganglia, 83.3% (30/36) comprised inferior cervical and first thoracic ganglia, with a single case (2.8%) incorporating the second thoracic ganglion as well [42].

Filion and Lamb (2023) found that an inferior cervical ganglion coalesced with the T1 ganglion in just 37.29% of specimens [23]. A fusion extended also to the T2 ganglion, determining a “stellate segment”, was encountered in 49.15% of the sample [23]. The bilateral prevalence of the fused inferior cervical and first thoracic ganglia was 31.82% [23]. On the other hand, all of the 59 SG studied by Samrid et al. (2024) [3] were a fusion between the inferior cervical and the first thoracic sympathetic ganglia. No contributions from the second thoracic sympathetic ganglion were identified (no stellate segment) [3].

**Table 1:** Anatomical variations of stellate ganglion, clinical implications, and imaging recommendations.

Variation	Prevalence	Clinical Impact	Imaging Recommendation	Key References
**Ganglion Formation**				
True fusion (C7 + T1)	37.29–100%	Standard block approach	Ultrasound at C6-C7	[1,3,23,42,47]
Separate ganglia	14–20%	May require dual injection	MRI for planning	[4,42]
Includes T2–T4	3–10%	Extended block needed	Consider CT guidance	[42,45]
**Associated Structures**				
Nerve of Kuntz present	33–68%	Risk of incomplete block	Thoracoscopic evaluation	[19,22,26]
Intermediate/vertebral ganglion	60–94%	Additional target	High-resolution ultrasound	[20,41,42]
Multiple cords of the subclavian ansa	10–15%	Complex neural pathways	MRI neurography	[48,49]
**Vascular Relations**				
Aberrant vertebral artery	8–10%	High complication risk	Mandatory ultrasound with Doppler	[27,30,34,50]
Anterior vertebral artery at C6	>90%	Injection hazard	Color Doppler essential	[34,51]
Variant inferior thyroid artery	5–7%	Hematoma risk	Pre-procedural vessel mapping	[31,52,53,54]
Ascending and deep cervical arteries	N/A			[30,50,55,56]
**Positional Variations**				
Supracostal position	Variable	Standard approach effective	Lateral neck radiograph	[46,57]
Intrathoracic position	25% (left > right)	Risk of pneumothorax	CT or fluoroscopy	[43,57]
Perforated morphology	14.5%	Incomplete block possible	High-resolution imaging	[47]

The SG lies anterior to the neck of the first rib (thus lateral to the head of the first rib) and to the T1 nerve (Figure 1), but it variably ascends to the transverse process of the seventh cervical vertebra [43]. It may be lowered, thus being located posterior to the pleura and anterior to the first intercostal space, reaching the 2nd rib [43]. It reaches the 2nd rib in 25% of cases, more frequently on the left [57]. According to Paturet (1964), the SG may have a cervical, supracostal position, or it may be lowered in an intrathoracic, infracostal position [57]. However, most frequently, the SG has an intermediate, precostal position, between the neck of the first rib, posteriorly, and the pleural dome, anteriorly [57]. Five main forms of SG were documented by dissections: spindle (31.9%), dumbbell (23.2%), truncated (21.7%), perforated (14.5%), and inverted-L (8.7%) [47].

The SG occupies the supra-retro-pleural fossa of Sebileau and Thoma Ionescu, postero-superiorly to the pleural dome (cervical pleura) [43,57,58,59] (Figure 1, Figure 2, Figure 3, Figure 4 and Figure S2). This fossa is limited as follows: (1) medially, by the vertebro-pleural ligament; (2) laterally, by the costo-pleural ligament; (3) inferiorly, by the pleural dome; (4) supero-laterally, by the vertebro-pleuro-costal ligament; (5) posteriorly, by the posterior end of the first rib [43].

The pleural dome separates anteriorly the SG from the subclavian artery [43]. Beheshti et al. (2017) indicated that the SG is located “inferior to the subclavian artery”, which is an erroneous anatomical information [60]. The only structure separating the ganglion from the pleura is Sibson’s fascia (suprapleural membrane) [61]. This proximity means the pleura is a critical anterior relation to the SG. The vertebral vessels course in front of the SG, and the costocervical trunk crosses the outer side of the SG [43]. The ganglion is covered by the prevertebral fascia, which is a layer of the deep cervical fascia [5]. Therefore, when injecting anesthetic for SGB, the solution may spread in two ways: either deep to the carotid sheath or deep to the prevertebral fascia [62]. Injections of local anesthetic superficial to the prevertebral fascia will not reach the SG [62].

The thoracic duct and SG are both located in the lower neck and upper thorax, on the left side, making their anatomical relationship important for clinicians performing interventions in this region. The thoracic duct is medially and slightly posterior to the SG (Figure 1). Understanding their proximity helps minimize complications during procedures such as SGB or thoracic duct embolization [63,64]. High-resolution MRI and CT can help distinguish the SG (anterior to C7 transverse process) from the thoracic duct (posterior and medial, near the venous jugulosubclavicular angle) [60,64]. Awareness of this anatomy is critical during SGB to avoid thoracic duct puncture, which can lead to chylothorax—a potentially serious complication [63].

Gunduz and Kenis-Coskun (2017) [65] were quoted by Millhouse et al. (2025) [66] when they listed aberrant details on the anatomy of the SG: “The stellate ganglion [...] is bounded anteriorly by the trachea and esophagus and medially by the thyroid cartilage and the transverse process of the C7 vertebral body. It is located deep to the sternocleidomastoid muscle, medial to the scalene muscles and anterolateral to the longus coli, in close proximity to many vascular structures including the vertebral and carotid arteries” [65,66]. However, Gunduz and Kenis-Coskun (2017) described just that the SG “is located as follows: medial to the scalene muscles; lateral to the longus colli muscle, esophagus, and trachea, along with the recurrent laryngeal nerve in between; anterior to the transverse processes; superior to the subclavian artery and the posterior aspect of the pleura; and posterior to the vertebral vessels at the C7 level” [65], which is anatomically adequate. Millhouse et al. (2025) discuss in their review that computed tomography and ultrasound guidance offer the advantage of imaging of other structures around the SG, “such as the thyroid cartilage and artery, [....]” [66], which is highly confusing and anatomically false.

The SG may consist of two distinctive parts joined by nerve trunks (Figure 2); the lower one is the SG proper, and the upper part is applied anteriorly or antero-medially onto the vertebral artery, being termed either the intermediate ganglion (IG) or the vertebral ganglion [21,43,45,67,68]. Some authors located the IG behind the pre-foraminal segment of the vertebral artery (inferior to its entry in the C6 transverse foramen) [69], such as it appears in Figure 1 and Figure 4. There is a balance between the volume of the SG and IG; when one is small, the other is large [43]. Thoma Ionescu (1923) [59], quoted by Axford (1928) [68], described the inconstant IG: when present, it lies antero-medially to the vertebral artery and is superior to the SG, and it may occur either in the presence or in the absence of a middle cervical ganglion [59,68]. The vertebral ganglion typically resides on the anterior aspect of the vertebral artery, frequently located at the junction of the anterior and posterior components of the subclavian ansa [42]. Like the middle cervical ganglion, it may supply grey rami communicantes to the fourth and fifth cervical spinal nerves [45]. The IG of Ionescu is regarded as the vasomotor centre of the larynx; thus, it should be kept in the anatomical nomenclature [70]. The vertebral ganglion is not infrequently fused with the middle cervical ganglion (Figure 4), thus forming a medio-vertebral cervical ganglion, as termed by Wrete in 1959 [4]. Mannu (1914) [71], quoted by Ren et al. (1993) [21], considered all ganglia between the superior cervical ganglion and the SG as IG; he distinguished as particularly characteristic a superior one, the thyroid ganglion, and an inferior one, close to the subclavian artery, the vertebral or subclavian ganglion [21,71]. The incidence of the vertebral ganglion varies from 33.3% to 94.7% among different studies [20,41,42]. Axford (1928), quoted by Manuel et al. (2025), termed the two ganglia found between the superior cervical ganglion and the SG as “high and low middle cervical ganglia” [20,68].

Siwe (1931) found several small ganglia immediately above the subclavian artery, but he did not term them [72]. A ganglion was found, exactly anterior to the vertebral artery; lateral to it was another ganglion, and immediately medial, in the course of the sympathetic trunk, one or two more ganglia, with an indistinct line of limitation between them [72]. The last ones mostly resembled, in size, position, and connections, a middle cervical ganglion [72]. Two subclavian ansae were found: a typical one between the medial ganglion and the SG, and an additional one between the lateral ganglion and the SG [72].

### 3.3. Connections of the Stellate Ganglion

The SG gives rise to multiple mixed branches, including the vertebral nerve, subclavian branches, and inferior cardiac nerve (Figure 1, Table 2). These branches contain both preganglionic and postganglionic axons, with some axons synapsing within the ganglion and others traversing it without synapse [73]. Notably, preganglionic axons can travel through the stellate ganglion to the vagus nerve, inferior cardiac nerve, and cervical sympathetic trunk, highlighting complex interconnections within the autonomic nervous system [74].

**Table 2:** Anatomical and clinical characteristics of branches of the stellate ganglion (SG) [40,41,42,43,45,47,70]. SG—stellate ganglion; IG—inferior cervical ganglion; CT—cervicothoracic ganglion (fusion of IG with T1 ganglion); MG—middle cervical ganglion; SN—superior cardiac nerve; IN—inferior cervical cardiac nerve; MN—middle cardiac nerve; TN—thoracic cardiac nerve.

Branch	Target/Destination	Characteristics	Clinical Significance	Key References
gray communicating rami	C7-T2 spinal nerves (or C8-T2).	Variable number: C7 has 1–5 rami (usually 2); the 3rd ramus may ascend medial to the vertebral artery, traverse C6 foramen with vertebral vessels; an inconstant ramus may traverse C7 foramen. C8 has 3–6 rami. T1 variable. The anterior scalene muscle is an essential relation.	Supply sympathetic innervation to corresponding dermatomes and myotomes.	[40,45]
vertebral nerve	C4-C7 spinal nerves (indirect); vertebral artery plexus.	Cranial deep communicating ramus; most commonly connects to C6-C7; it passes through transverse foramina.	Sympathetic innervation of the vertebral artery; possible role in the vertebrobasilar circulation; may be involved in certain headaches.	[75,76,77,78]
direct superficial branches	C6-T1 spinal nerves; occasionally C5.	Short connections; superficial course.	Direct sympathetic supply to the upper limb via brachial plexus.	[70]
T1 communicating branch	First thoracic nerve.	Contains myelinated fibers from ciliospinal nucleus; short and deep; courses on the pleural dome.	Physiological mydriasis pathway; damage causes Horner’s syndrome; vulnerable in neck surgery and Pancoast tumor.	[70,79]
phrenic nerve connection	Phrenic nerve.	Direct connection; almost constant.	Sympathetic influence on the diaphragm; may contribute to respiratory-autonomic integration.	[44,70,80]
vagal connections	Vagus nerve.	Almost constant connection; sometimes a direct branch from the SG.	Parasympathetic-sympathetic interaction.	[44,49]
recurrent laryngeal connection	Recurrent laryngeal nerve.	Almost constant connection.	Mixed motor, sensory, and sympathetic innervation of larynx.	[44,70]
superior cardiac nerve (SN)	Cardiac plexus/Heart.	Originates from: superior cervical ganglion (88.5%) or sympathetic trunk between SG and MG (71.2%).	Sympathetic cardiac innervation; accompanies great vessels (brachiocephalic trunk, common carotid arteries) to reach the heart.	[42]
inferior cervical cardiac nerve (IN)	Cardiac plexus/Heart.	Originates from the IG or SG; observed in 86.0%. Course: Descends behind the subclavian artery, along front of trachea to the deep cardiac plexus. Connections: Connects with the recurrent laryngeal nerve and cardiac branch of middle cervical ganglion (or replaced by fine branches from IG and ansa subclavia).	Principal cardiac branch from the SG; consistently present bilaterally; major contributor to the cardiac plexus.	[42,81,82]
middle cardiac nerve (MN)	Cardiac plexus/Heart.	Can originate from multiple sources: MG (87.8%), vertebral ganglion (86.0%), sympathetic trunk between MG and SG (76.9%). Includes contributions from the subclavian ansa.	Major cardiac contributor; one of the main sympathetic pathways to the heart; considered a principal component of the human cardiac innervation.	[42]
thoracic cardiac nerve (TN)	Cardiac plexus/Heart.	Originates from thoracic ganglia or thoracic sympathetic trunk below SG; observed in 67.3%.	Complex course in the posterior mediastinum; right TN may follow a “recurrent” path along the thoracic aorta; left TN uses the aortic arch.	[42]
subclavian artery plexus	Subclavian artery and branches.	Direct vascular branches from the SG to nearby vessels.	Vasomotor control of subclavian territory.	[19,43]
brachial plexus pathway	Axillary artery (via brachial plexus).	Indirect vascular branches for the upper limb.	Sympathetic vasomotor control of the upper extremity.	[19,70]
internal thoracic artery branch	Internal thoracic artery.	May receive phrenic nerve contribution.	Vascular sympathetic supply.	[45]
inferior thyroid artery plexus	Thyroid gland; recurrent laryngeal nerve; external laryngeal nerve; common carotid plexus.	Connects with multiple neural structures.	Complex autonomic-endocrine integration.	[45]

#### 3.3.1. The Communicating Branches of the SG

The SG generally provides gray communicating rami for the 8th cervical to the 2nd thoracic segmental levels [44]. According to Potts (1925), it supplies communicating branches to the 7th and 8th cervical, and 1st thoracic nerves [40]. The anterior scalene muscle presents an essential relation to the gray communicating branches; the 5th and 6th or 7th nerves may lie in front of the muscle, may pierce it, or may lie under the cover of the muscle [40]. So that in some cases in which these nerves lie upon, or pierce the anterior scalene muscle, it is not uncommon to find all the rami, or some of them, lying upon the surface of the muscle, or wandering among its fibres [40].

The number of the gray communicating branches of the SG is variable [45]. The cranial deep communicating ramus is the vertebral nerve [4]. It connects constantly with the phrenic nerve and almost as often with the vagus and recurrent laryngeal nerves [44,70]. Inferior cervical sympathetic cardiac nerves, variable in number and connections, arise from the SG and the ansa subclavia [44] (Figure 1, Figure 2, and Figure 4). According to Rouviere and Delmas, the SG is united directly to the C7-T1 nerves, the middle cardiac nerve, and the phrenic nerve, and communicates indirectly, via the vertebral nerve, with the C4-C7 nerves [43]. According to Delmas and Laux, the SG has direct (superficial) communicating branches that connect to the C6-T1 nerves and, occasionally, the C5 nerve [70]. As these authors discussed, the communicating branch with the first thoracic nerve is physiologically different: it contains myelinated fibers from the ciliospinal nucleus (indicated by the authors as “spinal oculo-pupillary center”) [70]. The communicating branch with the T1 nerve is short and deep into the supra-retro-pleural fossa, thus it courses on the pleural dome [70]. As this sympathetic pathway is responsible for physiological mydriasis, Horner’s syndrome may result when the communicating branch with the T1 nerve is damaged in surgeries of the neck or bronchogenic carcinoma (Pancoast tumor) [79]. While direct vascular branches of the SG supply the plexus of the subclavian artery and its branches, the vascular branches for the upper limb reach the axillary artery via the brachial plexus and the respective communicating branches [70]. A branch of the phrenic nerve may join the extension of the subclavian plexus to the internal thoracic artery [45]. The plexus on the inferior thyroid artery reaches the thyroid gland and connects with the recurrent and external laryngeal nerves, the cardiac branch of the superior cervical ganglion, and the common carotid plexus [45].

#### 3.3.2. The Subclavian Ansa of Vieussens

**Figure 5:**
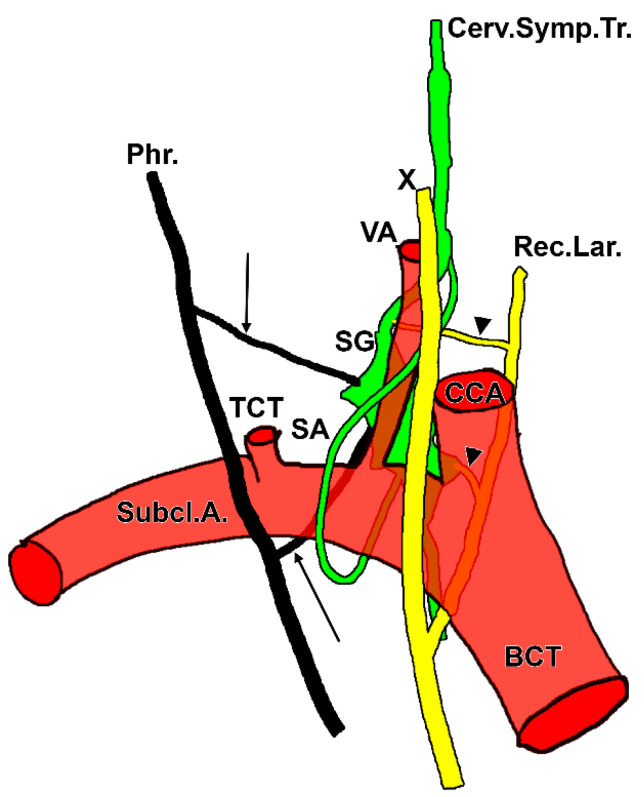
Schema depicting the classic nerve loops beneath the right subclavian artery. Anterior view. Phr: phrenic nerve; X: vagus nerve; Cerv.Symp.Tr.: cervical sympathetic trunk; Rec.Lar.: recurrent laryngeal nerve; BCT: brachiocephalic trunk; CCA: common carotid artery; Subcl.A.: subclavian artery; VA: vertebral artery; TCT: thyrocervical trunk; SG: stellate ganglion; SA: subclavian ansa. The arrows indicate the anastomoses between the phrenic nerve and the cervical sympathetic trunk. The arrowheads indicate the sympatho-recurrential anastomoses.

In just 10% of cases, three nerve loops simultaneously cross beneath the subclavian artery (Figure 5): the ansa subclavia (AS), the anastomotic ansa between the recurrent laryngeal nerve and the cervical sympathetic trunk, and the anastomotic ansa between the phrenic nerve and the SG [48].

**Figure 6:**
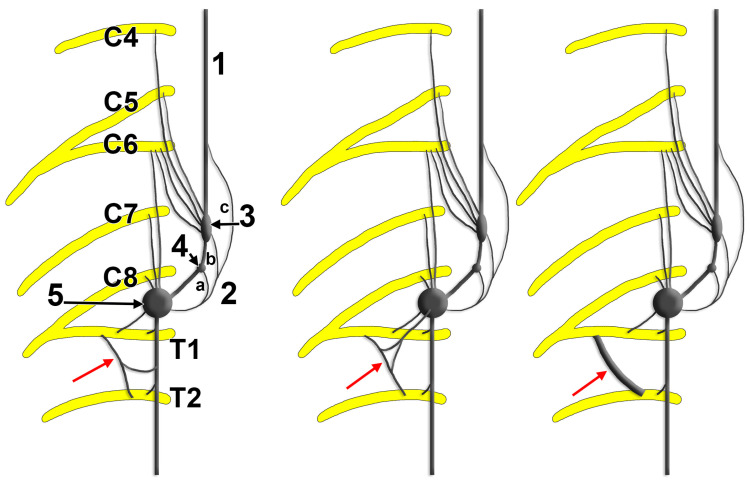
Schema of the morphological variants of the intrathoracic nerve (red arrows) described by Küntz (1927) and of those of the ansa subclavia depicted by Paturet (1964) [57,98]. 1. Sympathetic trunk; 2. ansa subclavia: short, connected to the vertebral ganglion (a), short, connected to the middle cervical ganglion (b), long (c); 3. middle cervical ganglion; 4. vertebral ganglion; 5. stellate ganglion.

The AS (Figure 1, Figure 2, Figure 3, Figure 4, Figure 5 and Figure 6) is a nerve cord that connects the middle cervical and inferior cervical sympathetic ganglia and forms a loop around the prescalenic segment of the subclavian artery [49,83]. For other authors, the AS unites both parts of the SG, superior and inferior [48], a morphology we did not encounter in our dissection studies. The AS may send off cardiac branches [83].

The anterior arm of the ansa was termed the ventral ansa, while the posterior arm of it was termed the dorsal ansa [81]. The dorsal ansa may contain one or more ganglia, and in some instances is composed of a continuous band of neuronal cell bodies [81]. The AS crosses the prescalenic segment of the subclavian artery between the origin of the vertebral artery, medially, and those of the thyrocervical trunk and internal thoracic artery, laterally.

The AS may appear as single or multiple cords, and the right side has more nerve cords in total than the left [49]. The significance of this difference is unclear [49].

Paturet (1964) [57], quoted by Caliot et al. (1984) [48] and by Loukas et al. (2008) [49], described different types of AS, as follows: “on the left, a single, flattened, ribbon-like loop is observed, placed like a lamella surrounding the artery. From this ansa a flattened filament, variable in length, descends to encircle the outer and anterior sides of the thoracic portion of the subclavian artery to merge with the lower cardiac nerve; this is Guillaume’s small spiral band; on the right, it is only in exceptional cases that the ansa is single; it usually consists of two to five flattened, contiguous filaments, placed on the anterior side of the artery, at a width of 5 to 6 mm” [48,49,57].

The AS forms the connection between the middle cervical ganglion and the SG [49]. The middle cervical ganglion or the AS are anastomosed with the recurrent laryngeal nerve and the phrenic nerves, respectively [49]. In addition, there is an evolutionarily conserved direct connection between the AS and the vagus nerve [49].

An anatomical and functional specificity of the cardiac innervations exists: electrical stimulation of the left cardiac sympathetic nerves results predominantly in pronounced changes in inotropic state, while stimulation of the right cardiac sympathetic nerves results predominantly in pronounced changes in chronotropic state of the heart [84]. The stimulation of the AS produces significant increases in the maximum rate of pressure development, heart rate, and blood pressure [49].

#### 3.3.3. The Recurrent—Sympathetic Anastomosis

A direct anastomosis (communicating branch) between the recurrent laryngeal nerve and the cervical sympathetic trunk is a recognized but relatively rare anatomical variant. This connection most commonly arises from the middle cervical sympathetic ganglion or directly from the sympathetic trunk and joins the recurrent laryngeal nerve, typically on the right side. This branch is sometimes called the sympathetic-inferior laryngeal anastomotic branch [85,86]. The anastomosis may be thin or, in rare cases, as large as the recurrent laryngeal nerve itself, which can lead to confusion during surgical procedures, especially thyroid or parathyroid surgery [85,86]. Histological and immunohistochemical studies confirm that the recurrent laryngeal nerve can contain sympathetic postganglionic fibers, which are likely contributed via these anastomoses [87,88]. Awareness of this anastomosis is crucial during neck surgery to avoid mistaking the sympathetic-inferior laryngeal anastomotic branch for a non-recurrent recurrent laryngeal nerve, which could result in inadvertent nerve injury [85,86]. These connections may influence the mixed motor, sensory, and sympathetic innervation of the larynx, potentially affecting laryngeal function and recovery after nerve injury [87,88].

#### 3.3.4. The Phrenic-Stellate Ansa

The anastomotic ansa between the phrenic nerve and the SG (phrenic-stellate ansa) is a rare anatomical variant, occasionally forming a neural loop beneath the subclavian artery (Figure 5 and Figure S1). Such a phrenic-stellate ansa has been identified in anatomical studies, but it is uncommon. In a series of 60 dissections, this ansa was present in only a minority of cases, and the classic configuration—where this loop coexists with other nerve loops beneath the subclavian artery—was found in just 10% of specimens. The connection is more rarely observed than other well-known nerve loops in the region [48]. While the direct functional significance of this ansa is not fully established, its presence may have implications for (a) surgical procedures in the lower neck and thoracic inlet, where unexpected neural connections could increase the risk of nerve injury; (b) autonomic and respiratory integration, as the phrenic nerve is the main motor supply to the diaphragm and the SG is a major sympathetic ganglion. However, no direct evidence links this ansa to specific clinical syndromes or functional outcomes [48].

Anatomical studies confirm that the phrenic nerve can receive sympathetic fibers from cervical sympathetic ganglia, including the SG, and that communicating fibers exist between these structures [80]. The phrenic nerve may also communicate with the subclavian ansa and the splanchnic nerves [80]. However, these connections are primarily autonomic, and their direct impact on diaphragmatic motor function is not established [80,89]. The sympathetic fibers coursing through the phrenic nerve are TH-positive catecholaminergic fibers and are supposed to be vasoregulators of the diaphragmatic vessels [80].

#### 3.3.5. The Vertebral Nerve

The VN provides sympathetic innervation to the vertebral artery (causing vasoconstriction), contributes to the regulation of blood flow to the brainstem and cerebellum, and thus may play a role in vertebrobasilar circulation. It can be involved in certain types of headaches and may be affected by cervical spine disorders. It is sometimes considered in treatments for certain vascular conditions affecting the posterior circulation.

There is a significant paucity in the literature regarding the vertebral nerve [78]. The vertebral nerve is named after the French physiologist Charles Émile François-Franck [76,78,90,91]. It arises from the SG (Figure 1, Figure 2, Figure 3 and Figure 4) and travels along the vertebral artery through the transverse foramina of the cervical vertebrae. The vertebral nerve appears to be a complex of communicating branches to the C5-C7 cervical spinal nerves [77]. Fine filaments from the vertebral nerve and the gray communicating rami form a vertebral plexus on the surface of the vertebral artery. The vertebral nerve is regarded as a macroscopically discernible nerve and is distinct from any microscopic adventitial plexus of the vertebral artery [75]. To this plexus may also contribute the vertebral ganglion. This plexus contains not only sympathetic efferent fibres but also somatic sensory fibres from the adventitia of the artery, with their cell bodies in the cervical dorsal root ganglia [45]. The vertebral nerve sends filaments to the cervical intervertebral discs and gives off meningeal branches (sinuvertebral nerves) at each cervical segment [45]. The meningeal branches are occasional [78]. According to Gray’s Anatomy, the vertebral plexus contains some neuronal cell bodies and extends along the vertebral and basilar arteries and their branches as far as the posterior cerebral artery, where it meets a plexus from the internal carotid artery [45].

However, Hollinshead (1982), quoted by Tubbs et al. (2007), observed that the vertebral nerve “does not follow the vertebral artery into the skull, but is replaced in the upper part of the neck by another plexus the sensory fibers of which are derived from the third or second and third spinal nerves and whose sympathetic fibers arise from the superior cervical ganglion” [78,92]. The V3 and V4 segments of the vertebral artery may be supplied by filaments from the C1 and C2 ventral rami, and from the hypoglossal nerve [75]. Physiologically, the vertebral artery is minimally responsive to stimulation of either the vertebral nerve or the sympathetic trunk; therefore, irritation of the vertebral nerve could not be a cause of cervical or vertebrobasilar migraine [75].

The sinuvertebral nerve was presented slightly differently by Xiuqing et al. (1988) [69]. They described that this nerve results after the anastomosis of a ramus from the vertebral nerve and the “corresponding spinal branch of the third to the sixth cervical nerves”. The authors detail that the sinuvertebral nerve averages 0.3 mm in diameter, it enters the vertebral canal and ascends in the posterior longitudinal ligament, supplying branches to the articular capsules of Luschka’s joints, the spinal dura mater, the posterior longitudinal ligament, the annulus fibrosus, and vertebral bodies [69].

Yan et al. (2009) considered, after a dissection study, that the vertebral nerve and the fibres surrounding the vertebral artery could be regarded as a stable deep pathway of cervical sympathetic nerves [93]. They considered that this deep pathway, together with the superficial one (cervical sympathetic trunk and its branches), forms a sympathetic nervous “plexus” in the neck [93]. Indeed, as Hoffman and Kuntz (1957) reported, from a surgical point of view, it is evident that complete sympathetic denervation of the upper extremity and the cervical and cephalic regions cannot be achieved without interruption of the vertebral nerve (quoted in Johal et al., 2017) [76,94].

The vertebral nerve has been variably described as arising from the SG, inferior cervical ganglion, middle cervical ganglion, vertebral ganglion, or from the ansa subclavia [78]. A difference in the origin of the vertebral nerve between fetal and adult cadavers was noted [76]. It may be located within a narrow triangle bordered medially by the longus colli muscle, laterally by the vertebral artery, and inferiorly by the neck of the first rib [40,68]. It may, however, ascend postero-laterally to the vertebral artery, therefore outside of this triangle (Figure 2 and Figure 4). The triangle of the vertebral nerve should not be confused with the triangle of the vertebral artery, which is bounded by the anterior scalene muscle laterally, the longus colli muscle medially, and the subclavian artery inferiorly [95].

The vertebral nerve appears as a long and deep gray communicating branch that connects most commonly the SG to C6 and/or C7 spinal nerves and courses through the C6 and C7 transverse foramina [72,78]. Siwe (1931) nicely described that the vertebral nerve runs up in a reasonably straight line and crosses the vertebral artery dorsally from the lateral to the medial side to join the C6 or C7 nerve [72]. In a few cases, it was found to divide and give a branch for each of these nerves [72]. Siwe (1031) found on the vertebral artery just a vascular plexus derived from the plexus on the subclavian artery, which was traced as far as the base of the skull [72].

Van den Broek (1908), quoted by Siwe (1931), described it as being formed in man by communicating branches from the 6th, 7th, and 8th cervical nerves [72,96]. Potts (1925), as quoted by Hoffman and Kuntz (1957), described the vertebral nerve as a constant gray communicating branch associated mainly with the 7th cervical nerve [40,94]. Yan et al. (2009) found in 36/36 sides that the C4-C7 cervical nerves received one or two branches of the vertebral nerve, and the terminal segment of the vertebral nerve was found to be at C3 in most cases [93]. The vertebral nerve is plexiform in 15% of sympathetic trunks [78]. In 50%, it has small branches that enter the fibrous capsule of adjacent zygapophyseal and intervertebral joints [78].

Xiuqing et al. (1988) described that most branches of the SG distribute to the surface of the V1 segment of the vertebral artery and the vertebral nerve is the largest one [69]. The authors found in 20 cadavers that the vertebral nerve is unique in 90% and has a diameter of 1.6 ± 0.2 mm [69]. While slender branches of the vertebral nerve form the vertebral plexus around the vertebral artery, the larger branches of the nerve join the C7 spinal nerve [69]. The middle cervical ganglion and the sympathetic trunk adjacent to it contribute branches to the plexus of the vertebral artery [69].

Bogduk et al. (1981) dissected three embalmed human adult cadavers and concluded “there is no individual nerve which may be referred to as vertebral nerve” [75]. Instead, the V2 segment of the vertebral artery ascending through the transverse foramina of the cervical vertebrae is accompanied by a repeating system of neural arcades [75], which were regarded by Yan et al. (2009) as an “arched-shaped” fiber bundle on the ventral surface of the vertebral artery [93].

#### 3.3.6. The Subclavian Sympathetic Plexus

The subclavian plexus (Figure 1) is a network of sympathetic nerve fibers associated with the subclavian artery. Understanding its anatomy is crucial for surgical and interventional procedures in the neck and upper thorax. The subclavian plexus originates from the upper thoracic sympathetic trunk, particularly from the SG and the first few thoracic ganglia. It forms a plexus around the subclavian artery as the artery arches over the first rib and passes into the axilla. The plexus may also receive contributions from the middle cervical ganglion [19]. It innervates the subclavian artery and its branches, influencing the vasomotor tone in the upper limb and parts of the thorax. It also sends fibres to the brachial plexus, contributing to the sympathetic supply of the upper extremity [19]. Variations in the anatomy of the upper thoracic sympathetic chain, including the presence of the SG and nerve of Kuntz, can affect the distribution and clinical relevance of the subclavian plexus [19]. Knowledge of the subclavian plexus is essential for procedures such as sympathectomy, SGB, and surgeries involving the subclavian artery or the brachial plexus. Anatomical variations, such as the presence of accessory rami or variable ganglion positions, can impact the surgical outcomes and increase the risk of nerve injury [19,97]. Injury or interruption of these fibres can affect upper limb blood flow, sweating, and pain states.

#### 3.3.7. The Nerve of Küntz

The nerve of Küntz (intrathoracic nerve) is an anatomical variant of sympathetic innervation (Figure 6) that has significant clinical implications, particularly in thoracic sympathectomy procedures. It was described in 1927 [98]. The nerve of Küntz represents accessory sympathetic fibres that bypass the standard sympathetic chain pathway. These fibres typically originate from the T2 spinal nerve, directly to the SG or first thoracic ganglion, and may also connect the T2 and T3 ganglia [99]. They run intrathoracically, often along the posterior aspect of the first rib, lateral to the typically located sympathetic chain [99]. The nerve of Küntz can present in several configurations: (a) single communicating branch; (b) multiple small branches; (c) nerve plexus formation; (d) connections at different vertebral levels. Küntz’s nerve is an anatomical variant of the sympathetic nervous system, often implicated in surgical failures after sympathectomy. Its reported prevalence varies significantly. Cadaveric dissection studies commonly report prevalence rates between 33% and 68% [22,23,26]. For example, one study found the nerve in 66% of sides dissected, with bilateral presence in 78.9% of cases [26]. Another reported a 68.2% prevalence on dissected sides and bilateral presence in 48.1% of cadavers [22]. Recent anatomical reviews estimate the average prevalence to be approximately 53% [19].

The detection method matters: thoracoscopic (surgical) identification rates are much lower (as low as 12%) compared to open anatomical dissection (up to 66.7%) [24,25]. This discrepancy is likely due to the small size and low color contrast of the nerve, making it difficult to visualize during surgery [24]. Population and sex differences: some studies note a higher prevalence in males and variability between right and left sides, but findings are inconsistent [23].

#### 3.3.8. The Visceral Branches of the SG

The SG provides direct visceral branches to the pleural dome, trachea, and esophagus of minor physiological importance, while cardiac branches represent the most clinically significant output (Delmas & Laux 1933 [70]). Most sympathetic cardiac innervation from the SG is via the middle and inferior cervical cardiac nerves (ICCNs) (Figure 1), which are clinically relevant for procedures like SGB or surgical denervation [82,100,101].

According to Kawashima’s comprehensive anatomical studies [42,77], the ICCN is the principal cardiac branch from the SG, consistently present bilaterally, and represents a major contributor to the cardiac plexus. The works of Kawashima [42,77] should be observed. The ICCN often receives additional fibers from the ansa subclavia. The right ICCN descends along the brachiocephalic artery’s posterior aspect, while the left follows the left common carotid artery, both crossing posterior to the aortic arch to reach the deep cardiac plexus [42,81].

Cardiac sympathetic postganglionic fibers originate from the SG, T2-T4 ganglia, middle cervical ganglia, subclavian ansa, and, to a lesser extent, the superior cervical ganglia [83,102]. All major sympathetic cardiopulmonary nerves arise from the SG and caudal cervical sympathetic trunks below the cricoid cartilage [81]. These mixed neural pathways integrate sympathetic and parasympathetic influences, with some branches containing preganglionic fibers traversing the SG [74,103]. Small ganglia may exist along cardiopulmonary sympathetic nerves [81,104].

Laterality differences exist: right cardiac nerves robustly innervate atria, including sinoatrial and atrioventricular nodes, affecting chronotropic state, while left sympathetic trunk predominantly affects ventricular contraction and inotropic state, though interspecies differences exist [102]. This laterality underlies different electrophysiological effects, with left SG stimulation enhancing heterogeneities more than right [102,105,106].

#### 3.3.9. Functional Influences of the Stellate Ganglion

In experiments with pseudorabies virus injections of the superior cervical ganglion or SG, cell body infections were found in the mesencephalic central gray matter and the lateral hypothalamic area [107]. A much larger number of infected cells was seen in the lateral hypothalamic area LHA after SG experiments: the ventral zona incerta region was labeled only after SG injections [107]. These uniquely infected cell groups may subserve specialized functions: the central gray matter is a critical site coordinating the defense reaction, and the ventral zona incerta is involved in the regulation of heart rate [107].

Experiments have demonstrated the physiological role of a dual sympathetic (stellate and superior cervical) innervation in the control of cochlear blood flow [108].

Two distinct sympathetic components have been identified in the vestibular ganglion: a perivascular system derived from the SG, and a blood vessel-independent system derived from the superior cervical ganglion [45]. Vestibular responses elicited by electrical stimulation of the SG and of the vertebral nerve were observed in cases of intermittent vertebral artery compression (Powers’ syndrome) [109]. Stimulation of the vertebral nerve produced many varieties of pupillary change with or without ocular movements, nystagmus, posterior headache, and dizziness [109]. Stimulation of the SG produced just prompt and full mydriasis of the ipsilateral pupil [109]. Therefore, it seems likely that the vertebral nerve and plexus (“deep or posterior cervical sympathetic system”) is functionally different from the cervical sympathetic system, including the SG (“anterior cervical sympathetic system”) [109].

### 3.4. Imaging Anatomy of the SG

CT-guided SG injection is highly effective in achieving sympathetic blockade with a reduced anesthetic volume compared to conventional methods [110]. CT imaging provides precise anatomical details, improving both safety and efficacy compared to traditional landmark-based approaches. Clinicians and radiologists should, however, be aware that the neurovascular structures within the superior thoracic outlet are not bilaterally symmetrical or consistently distant as they appear on various schematic drawings of horizontal sections of the neck [111].

Key CT landmarks for SG localization are the transverse processes of C7 and C6, the cricoid cartilage, eventually, and the vertebral gutter between the lateral margin of the vertebral body and the transverse process (Table 3). The carotid tubercle of Chassaignac (the prominent anterior tubercle of the transverse process of the sixth cervical vertebra) is commonly used as a reference point, but the SG itself lies 1–2 cm lower, typically at the level of the C7 transverse process and the neck of the first rib [112,113]. The carotid tubercle is identified easily by locating the cricoid cartilage [113]. The cricoid cartilage often aligns with the C6 vertebral level in neutral position, but moves cephalad with neck extension. This variability means the cricoid cartilage is a less reliable landmark in extended positions, though it can still guide initial localization [112,113,114]. The SG is usually situated anterior to the C7 transverse process and superior to the neck of the first rib [60]. Needle placement for block is often safest where the transverse process joins the vertebral body, as this provides a larger bony target and reduces risk to adjacent structures [114]. Injections are made at the C6 on the C7 vertebra to diminish the risk of vascular or pleural damage [110].

**Figure 7:**
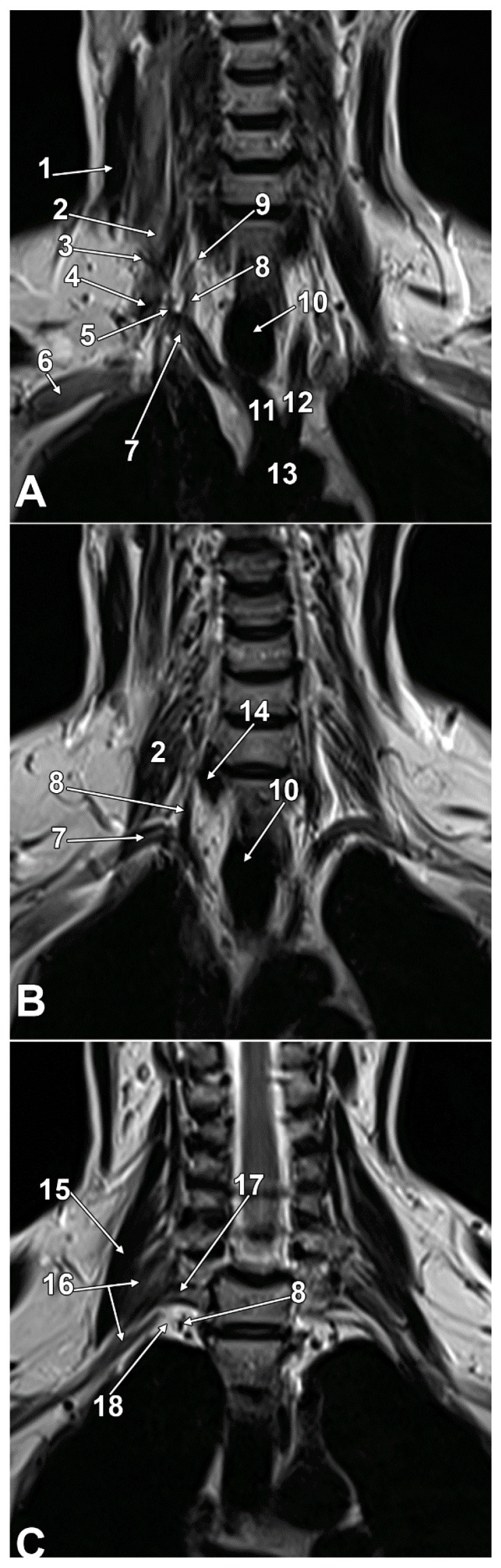
Successive anterior-to-posterior (**A**–**C**) original coronal MRI slices through the cervicothoracic region. Anterior view. Magnetic resonance imaging was performed using a 1.5 Tesla MRI scanner (Siemens Magnetom Avanto). Coronal plane T1-weighted turbo spin-echo imaging of the cervicothoracic junction was acquired with the following parameters: sequence: T1-weighted turbo spin-echo (TSE), repetition time (TR): 550 ms, echo time (TE): 12 ms, slice thickness: 3 mm, inter-slice gap: 0.3 mm, FOV: 300 mm, matrix: 384 × 384. Images were displayed using standard soft tissue window settings optimized for visualization of neural structures, muscles, and vascular anatomy. The stellate ganglion (18) was identified as an intermediate signal intensity structure at the thoracic inlet, located anterior to the neck of the first rib (17), lateral to the longus colli muscle (14), and posterior to the vertebral artery (8). The ganglion’s position relative to surrounding vascular structures (subclavian artery, vertebral artery) and bony landmarks (C7 transverse process, first rib) was clearly delineated. 1. sternocleidomastoid muscle; 2. anterior scalene muscle; 3. transverse cervical artery; 4. suprascapular artery; 5. thyrocervical trunk; 6. axillary vein; 7. right subclavian artery; 8. vertebral artery; 9. inferior thyroid artery; 10. trachea; 11. brachiocephalic trunk; 12. left common carotid artery; 13. aortic arch; 14. longus colli muscle; 15. middle scalene muscle; 16. posterior scalene muscle; 17. neck of the first rib; 18. stellate ganglion.

**Figure 8:**
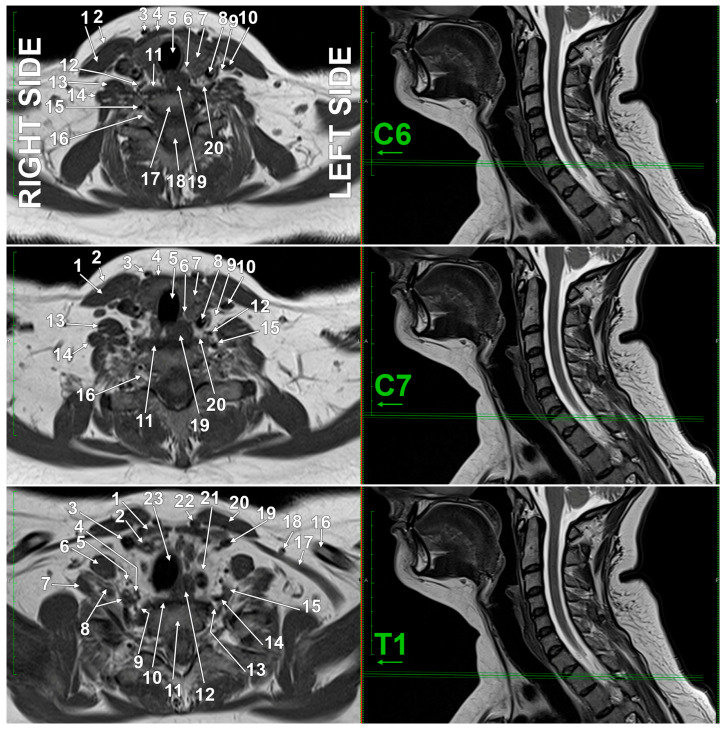
Sequence of original axial T2-weighted MRI slices through the C6, C7, and T1 vertebrae. Inferior views. The vascular topography on axial slices shows bilateral asymmetry. The scanning parameters were detailed in the previous legend. The C6 and C7 slices are similar; in these slices were identified the sternocleidomastoid muscle (1), platysma muscle (2); anterior jugular vein (3); strap muscles (4); trachea (5); recurrent laryngeal nerve (6); thyroid lobe (7); common carotid artery (8); vagus nerve (9); internal jugular vein (10); longus colli muscle (11); inferior thyroid artery (12); anterior scalene muscle (13); middle scalene muscle (14); vertebral artery (15); spinal nerve (16); vertebral body (17); spinal cord (18); esophagus (19); and the sympathetic trunk (20). At T1 there are identified the strap muscles (1); right common carotid artery (2); right internal jugular vein (3); right vertebral artery (4); right thyrocervical trunk (5); right anterior scalene muscle (6); right middle scalene muscle (7); right subclavian artery (8); right sympathetic trunk/stellate ganglion (9); longus colli muscle (10); vetebral body (11); esophagus (12); left sympathetic trunk/stellate ganglion (13); left vertebral artery (14); left subclavian artery (15); clavicle (16); omohyoid muscle (17); external jugular vein (18); left internal jugular vein (19); strap muscles (sternohyoid, sternothyroid) (20); left common carotid artery (21); anterior jugular vein (22); trachea (23).

Sympathetic structures such as the stellate and thoracic sympathetic chain ganglia can be clearly visualized using MRI, which provides excellent soft-tissue contrast and spatial resolution for identifying their anatomy and location [64]. So the SG may be clearly identified on MRI scans (Figure 7 and Figure 8) due to the excellent soft-tissue contrast and the possibility of direct multiplanar views [36]. Failure to correctly identify the SG could lead to misinterpretation on MRI scans of the complex regional anatomy [36]. Visualization of the SG via MRI has not been fully utilized, presumably due to the vagueness of its topographic anatomy, small size, suboptimal image quality, and limited reproducibility [37]. MRI consistently identifies the sympathetic trunk/SG at the thoracic inlet, adjacent to the neck of the first rib, lateral to the longus colli muscle, and posterior to the vertebral artery. The SG’s shape and position can vary, but it is reliably visualized in normal individuals using high-resolution MRI sequences [36,38]. The ganglion is typically located anterior to the transverse process of C7, superior to the neck of the first rib, and inferior to the subclavian artery [35]. Modern magnetic resonance imaging neurography, coupled with thorough anatomical knowledge, provides opportunities to precisely locate, characterize, and quantify SG morphology [37]. Maintaining interdisciplinary dialogue between anatomists, relevant clinicians, and radiologists will optimize the clinical application of these evolving imaging techniques [37].

**Table 3:** CT landmarks and their relation to the stellate ganglion (SG).

Landmark/Structure	Relationship with the SG	Clinical Note	References
C6 transverse process	1–2 cm above the SG	common needle entry, not SG level	[112,113,114]
C7 transverse process	directly posterior to the SG	true anatomical level of the SG	[60]
cricoid cartilage	variable with movement	useful in a neutral position, less in extension	[112,113,114]
neck of the first rib	inferior to the SG	lower boundary of the SG	[60]

## 4. Histological Organization of the Stellate Ganglion

The SG exhibits typical sympathetic ganglion architecture characterized by clustered postganglionic neurons surrounded by satellite glial cells (SGCs) within a fibrous capsule (Figure S3). The sympathetic neurons and SGCs communicate reciprocally [115]. The postganglionic neurons are large multipolar cells (20–50 μm diameter) containing prominent nuclei, Nissl bodies, lipofuscin pigment, and dense-core vesicles with norepinephrine. Preganglionic fibers from spinal segments T1–T4 form synapses primarily on dendrites and cell bodies using acetylcholine, while unmyelinated postganglionic C-fibers emerge to innervate target organs.

SGCs form continuous cellular envelopes around individual neurons, creating distinct functional units that regulate the neuronal microenvironment [116,117]. Unlike sensory ganglia, sympathetic ganglia contain synapses where SGCs overlay synaptic contacts, allowing for the modulation of transmission [117]. Recent transcriptomic studies have identified six SGC subtypes in the SG, each with distinct gene expression profiles, suggesting dynamic adaptation capabilities [118,119]. SGCs are extensively interconnected by gap junctions containing connexin 43, allowing coordinated responses that increase significantly following injury or inflammation [120,121,122,123].

SGCs serve multiple regulatory functions, including metabolic support through glucose and lactate transport [124,125], neurotransmitter clearance via specific transporters [126,127,128], and ionic homeostasis, particularly for potassium regulation. They express store-operated calcium entry machinery (Orai1/STIM1), which is critical for maintaining Ca^2+^ homeostasis, with disruption potentially affecting sympathetic output [115]. Additionally, sympathetic neurons directly sense ionic changes through Na^+^-sensitive Nax channels, contributing to sympathetic excitability [129,130].

Small intensely fluorescent (SIF) cells represent specialized chromaffin-like cells scattered throughout the SG that modulate ganglionic transmission. These neural crest-derived cells (10–20 μm diameter) contain dense-core vesicles with catecholamines and other neurotransmitters, including serotonin, bombesin/GRP, and enkephalins, with expression patterns varying by location and development [131,132]. Fenestrated capillaries are consistently found near SIF/SGC clusters rather than principal neurons, suggesting specialized exchange functions, while most neuronal capillaries maintain a restrictive blood-ganglion barrier [133,134,135].

Following injury or stress, SGCs become activated, characterized by GFAP upregulation, increased gap junction coupling, and cytokine release affecting neuronal excitability [117,136]. However, evidence for SGC proliferation in sympathetic ganglia remains limited compared to sensory ganglia, with apparent increases potentially representing GFAP upregulation or macrophage infiltration rather than true proliferation [136,137]. SGCs release neurotrophic factors and ATP that augment cholinergic transmission and promote synapse formation [117,138].

The SG receives arterial supply primarily from branches of the costocervical trunk, particularly the superior intercostal artery, with additional contributions from the inferior thyroid, internal thoracic, and first intercostal arteries [139,140]. The microvasculature demonstrates high density, with greater perikaryal vessel numbers compared to other ganglia, reflecting elevated metabolic demands [139].

This complex cellular organization enables the SG to integrate and modulate sympathetic outflow to cardiac, vascular, and other target tissues, with dysfunction contributing to various autonomic pathologies.

## 5. Clinical Applications

### 5.1. The Stellate Ganglion Block

The SG block (SGB) is a sympathetic interruption technique that relieves pain and modulates autonomic function by blocking neural transmission at the cervicothoracic junction [6,7]. The procedure aims to interrupt sympathetic outflow to the head, neck, upper extremities, and heart, potentially improving blood flow and reducing noxious stimulation peripherally while decreasing central pain transmission [18,141]. Successful SGB induces ipsilateral Horner’s syndrome and increases regional blood flow without affecting cardiac output, though contralateral flow may also increase [142]. There are, however, clear contraindications to an SGB [73] (Table 4).

**Table 4:** Contraindications for stellate ganglion block (SGB) [73]. Relative contraindications require individualized risk-benefit assessment in consultation with appropriate specialists [73].

Type	Contraindication	Rationale
absolute	active anticoagulation therapy	the risk of hemorrhage if vascular structures are inadvertently punctured during needle placement
absolute	contralateral pneumothorax or pneumectomy	the procedure carries a risk of iatrogenic pneumothorax, so, if the opposite lung is already compromised, this could result in bilateral pulmonary compromise
absolute	recent myocardial infarction	the SGB interrupts cardiac sympathetic innervation (accelerator fibers), which may adversely affect cardiac function in the acute post-infarction period
relative	glaucoma	repeated SGBs have been reported to trigger or exacerbate glaucoma in susceptible patients
relative	significant cardiac conduction abnormalities	the blockade of upper thoracic sympathetic ganglia can worsen bradycardia by removing sympathetic compensation for impaired conduction

Technical approaches include paratracheal (most direct, fewer complications), anterolateral, lateral, and posterior/paravertebral routes [46]. Ultrasound guidance is strongly recommended over blind techniques, improving safety and efficacy by enabling real-time visualization of needle entry and caudal anesthetic spread [51,143]. The C6 level is preferred over C7 to avoid pleural puncture, though vertebral artery variations at C6 occur in approximately 10% of patients [34,51]. Volume limitation to 4–5 ml of 0.25–0.375% local anesthetic balances efficacy with minimizing spread to adjacent structures [14,18]. Using fluoroscopy, a needle may be accurately directed towards the carotid tubercle [53]. However, this bony landmark is a surrogate marker for the SG, generally being closer to the middle cervical ganglion [53].

During spine surgery, SGB improves postoperative outcomes, including reduced sore throat, enhanced sleep quality, and accelerated gastrointestinal function recovery following lumbar and thoracolumbar procedures [7,144]. Animal studies suggest neuroprotective effects through reduced neuroinflammation and promotion of autophagy in spinal cord neurons [145]. SGB may also reduce postoperative cognitive dysfunction by modulating neuroendocrine stress responses [146,147].

Major vascular structures at risk include the vertebral artery (most common, especially with anatomical variants), inferior thyroid artery, ascending/deep cervical arteries, common carotid artery, and subclavian artery [30,31,32,52]. The internal jugular vein lies in an anterior plane to the SG and is often visualized or compressed during ultrasound-guided procedures, with inadvertent puncture causing hematoma risk [28,148].

While traditionally termed “stellate ganglion block,” anatomical studies reveal that true SG fusion occurs in only 80% of individuals, making “lower cervical sympathetic block” more anatomically precise [18]. Contraindications include coagulopathy, anticoagulation therapy, local infection, and contralateral recurrent laryngeal or phrenic nerve dysfunction [18]. Table 5, Table 6, and Table 7 provide comprehensive evidence for indications, complications, and technical recommendations, respectively.

**Table 5:** Clinical applications of stellate ganglion block—comprehensive evidence summary. Evidence Levels: A = Multiple RCTs or systematic review/meta-analysis; B = Single RCT or multiple cohort studies; C = Case series, case reports, or expert opinion Abbreviations: VT/VF = ventricular tachycardia/fibrillation; CSD = cardiac sympathetic denervation; RFA = radiofrequency ablation; ICD = implantable cardioverter defibrillator; CRPS = complex regional pain syndrome; SR = systematic review; TENS = transcutaneous electrical nerve stimulation; PSQI = Pittsburgh Sleep Quality Index; CBT-I = cognitive behavioral therapy for insomnia; GAD = generalized anxiety disorder; SSRIs = selective serotonin reuptake inhibitors; SAH = subarachnoid hemorrhage; MCA = middle cerebral artery; HRT = hormone replacement therapy; PTSD = post-traumatic stress disorder; EMDR = eye movement desensitization and reprocessing; HBO = hyperbaric oxygen.

Indication	Evidence Level	Study Type (*n*)	Success Rate	Patient Selection/Contraindications	Alternative Treatments	Key References
CARDIAC ARRHYTHMIAS						
Electrical storm	Level B	Cohort studies (*n* = 147)	70–80% reduction in VT/VF	Failed antiarrhythmics, hemodynamically stable	Surgical CSD, catheter ablation	[8,9,10]
Refractory VT	Level B	Case series (*n* = 58)	60–75% ICD shock reduction	>3 ICD shocks/24 h despite medical therapy	Bilateral CSD, RFA of SG	[105,149]
PAIN SYNDROMES						
CRPS Type I/II	Level A	2 RCTs (*n* = 124), SR	70–85% pain reduction	Failed conservative therapy > 3 months	IV regional blocks, spinal cord stimulation	[6,150]
Phantom limb pain	Level B	Case series (*n* = 42)	65–70% at 2 months	Post-amputation > 6 months	Mirror therapy, gabapentin, TENS	[151,152]
Postherpetic neuralgia	Level B	Retrospective (*n* = 86)	60–70%	Pain > 3 months post-rash	Pregabalin, lidocaine patch, RFA	[153,154]
Post-mastectomy pain	Level B	RCT (*n* = 60)	65–75%	Chronic pain > 6 months post-surgery	Intercostal blocks, gabapentin	[16,153]
SLEEP DISORDERS						
Primary insomnia	Level B	3 RCTs (*n* = 186)	70–75% PSQI improvement	PSQI > 7, failed behavioral therapy	CBT-I, benzodiazepines, melatonin	[14,155]
Postoperative sleep	Level B	2 RCTs (*n* = 120)	65–70% quality improvement	Major surgery, no respiratory compromise	Dexmedetomidine, melatonin	[12,15]
Anxiety-related insomnia	Level B	RCT (*n* = 80)	68% improvement	GAD with insomnia component	SSRIs, benzodiazepines, CBT	[13]
CEREBROVASCULAR						
Vasospasm post-SAH	Level C	Pilot RCT (*n* = 40), series	60–65% MCA velocity reduction	Hunt-Hess grade II-IV, day 3–14	Triple-H therapy, nimodipine, angioplasty	[156,157,158]
Cerebral blood flow	Level C	Case series (*n* = 25)	Variable improvement	Refractory vasospasm	Intra-arterial verapamil	[159]
AUTONOMIC DYSFUNCTION						
Hot flashes (menopause)	Level B	SR (*n* = 245)	60–65% symptom reduction	Failed hormone therapy or contraindicated	HRT, SSRIs, gabapentin	[5]
Hyperhidrosis	Level C	Case reports (*n* = 18)	70–80%	Primary palmar, failed topicals	Surgical sympathectomy, botulinum toxin	[160]
EMERGING INDICATIONS						
PTSD symptoms	Level C	Open label (*n* = 42)	Variable (40–60%)	Treatment-resistant PTSD	Prazosin, EMDR, prolonged exposure	[17]
Sudden hearing loss	Level C	Case series (*n* = 35)	45–55% hearing improvement	<72 h onset, failed steroids	Intratympanic steroids, HBO	[161]
Vestibular migraine	Level C	Open label (*n* = 30)	60% vertigo reduction	>3 attacks/month	Propranolol, topiramate, vestibular rehab	[162]

### 5.2. Evidence-Based Indications

The SGB has evolved from a primarily pain management technique to a versatile intervention with expanding therapeutic applications. Traditional indications for chronic pain syndromes, particularly complex regional pain syndrome and neuropathic pain, remain well-established, with multiple randomized controlled trials demonstrating efficacy [6,154]. However, recent evidence supports broader applications in cardiac arrhythmia management, where the SGB effectively reduces ventricular tachyarrhythmia burden in patients with electrical storm refractory to conventional therapy [8,9,10]. Emerging indications include sleep disorders, with randomized trials showing significant improvements in both primary insomnia and postoperative sleep disturbance [14,15], and cerebrovascular conditions, particularly vasospasm following subarachnoid haemorrhage [158,163]. Additionally, preliminary evidence suggests potential benefits in autonomic dysfunction, including menopausal hot flashes [5] and post-traumatic stress disorder [17], though these applications require further validation. Table 5 summarizes the current evidence base, success rates, patient selection criteria, and alternative treatment options for each indication, providing clinicians with practical guidance for incorporating SGB into multimodal treatment approaches.

### 5.3. Technical Approach

Optimal SGB technique requires careful attention to procedural details that significantly impact both efficacy and safety. Ultrasound guidance has become the standard of care, demonstrating superior outcomes compared to blind or fluoroscopic techniques through real-time visualization of needle placement and vascular structures [30,51,143]. The C6 level approach is preferred over C7 to minimize pneumothorax risk, though practitioners must recognize that vertebral artery variations occur in approximately 10% of patients at this level [34,164]. Volume optimization studies support 4–5 ml of 0.25–0.375% local anaesthetic as the ideal balance between therapeutic efficacy and minimizing spread to adjacent structures [14,165]. Patient positioning with contralateral head rotation increases the distance from the carotid artery, thereby reducing the risk of vascular puncture [166,167]. Pre-procedural vascular mapping with color Doppler identifies anatomical variants that may alter the approach [31,32]. Table 6 provides comprehensive, evidence-based technical recommendations that integrate anatomical knowledge with procedural safety considerations to optimize outcomes while minimizing complications.

**Table 6:** Evidence-based technical recommendations. Abbreviations: INR = international normalized ratio; RLN = recurrent laryngeal nerve.

Parameter	Recommendation	Evidence Quality	Rationale	Key References
**Approach**				
Imaging guidance	Ultrasound preferred	High	Reduces complications, real-time visualization	[30,51,143]
Entry level	C6 > C7	Moderate	Avoids pleura, reduces pneumothorax	[18,55,164]
Needle angle	In-plane visualization	High	Real-time monitoring, vessel avoidance	[30,155]
Lateral vs. medial	Paratracheal preferred	Moderate	Shortest route, fewer complications	[18,46]
**Volume/concentration**				
Local anesthetic volume	4–5 ml	Moderate	Balances efficacy/spread	[14,18,165]
Concentration	0.25–0.375%	Low	Reduces motor block	[14,51]
Test dose	0.5–1 mL initial	Low	Detects intravascular injection	[29,168]
**Patient positioning**				
Head position	Contralateral rotation	Moderate	Increases carotid distance	[166,167]
Neck extension	Mild extension	Low	Improves visualization	[51,155]
**Patient selection**				
Anticoagulation	Hold if INR > 1.5	High	Reduces hematoma risk	[52,55]
Bilateral blocks	Avoid	High	Risk of bilateral RLN/phrenic palsy	[18,169,170]
Respiratory compromise	Relative contraindication	Moderate	Risk of phrenic palsy	[161,170]
**Monitoring**				
Aspiration	Every 3–5 ml	High	Detects vascular puncture	[29,30]
Horner’s syndrome	Expected sign	High	Confirms successful block	[142,171]
Vascular Doppler	Pre-procedure scan	Moderate	Identifies aberrant vessels	[31,34]

**Table 7:** Complications of stellate ganglion procedures and management. Abbreviations: US = ultrasound; LA = local anesthetic; INR = international normalized ratio; RLN = recurrent laryngeal nerve.

Complication	Incidence	Risk Factors	Prevention Strategy	Management	Key References
**Major Complications**					
Vertebral artery injection	<0.5%	Blind technique, C6 approach	US guidance, aspiration	Supportive care	[30,51,168]
Pneumothorax	0.5–1%	Deep needle, low approach	Limit depth, C6 level	Chest tube if >20%	[55,61,164]
Seizures (LA toxicity)	<0.1%	Intravascular injection	Aspiration, low volume	Benzodiazepines	[29,168]
Spinal/epidural block	<0.1%	Deep medial needle	Lateral approach, US guidance	Supportive care	[18,170]
**Minor Complications**					
Horner’s syndrome	90% (expected)	Successful block	N/A (desired effect)	Reassurance	[142,171]
Hoarseness (RLN block)	10–15%	Large volume, spread	Limit to 4–5 ml	Observation	[165,169,172]
Phrenic nerve palsy	5–10%	Lateral spread	Medial approach	Supportive	[161,170]
Hematoma	1–2%	Coagulopathy, vessel injury	Check INR/platelets	Compression	[31,52]
Esophageal puncture	Rare	Medial needle placement	US guidance	Conservative	[46,55]
Brachial plexus block	3–5%	Lateral/posterior spread	Precise needle placement	Observation	[3,18]
**Bilateral Block Risks**					
Bilateral RLN palsy	High risk	Bilateral procedure	Avoid bilateral blocks	Airway support	[18,169]
Bilateral phrenic palsy	High risk	Bilateral procedure	Single-sided only	Ventilatory support	[18,170]

### 5.4. Complications and Management

While the SGB is generally safe, understanding potential complications and their management is essential for optimal patient outcomes. Major complications remain rare (<1%) with ultrasound guidance, though vertebral artery injection represents the most serious risk, particularly with blind techniques or unrecognized anatomical variants [30,168]. Pneumothorax risk increases with deeper needle placement and C7 approach, occurring in 0.5–1% of cases [55,61]. Another significant complication of the SGB is the intraspinal injection [73]. Minor complications are more frequent but typically self-limited, including expected Horner’s syndrome, confirming successful block in 90% of cases [142,171], transient hoarseness from recurrent laryngeal nerve involvement in 10–15% [165,172], and phrenic nerve palsy in 5–10% [161,170]. Bilateral procedures carry prohibitive risks of simultaneous bilateral recurrent laryngeal or phrenic nerve blockade, potentially causing respiratory compromise [18,169]. Vascular complications, including hematoma formation, are minimized through appropriate patient selection, particularly regarding anticoagulation status [31,52]. Table 7 provides comprehensive guidance on complication incidence, risk factors, prevention strategies, and management approaches, emphasizing that meticulous technique and appropriate imaging guidance significantly reduce adverse events while maintaining therapeutic efficacy.

## 6. Special Considerations

In hyperhidrosis surgery, the nerve of Küntz is a significant cause of incomplete sympathectomy results, recurrence of palmar hyperhidrosis after T2-T3 sympathectomy, or persistent sweating despite apparently successful surgery. Therefore, thoracic surgeons must actively search for and identify Küntz’s nerve during sympathectomy, divide any accessory pathways to ensure complete denervation, and extend the dissection to include potential variant pathways. During SG procedures, the incomplete blockade or the partial Horner’s syndrome after SGB may be explained by the presence of Küntz’s nerve. Understanding this variant helps explain why some patients may have asymmetric responses to bilateral procedures or an unexpected preservation of sympathetic function.

SG stimulation modulates cardiac function and autonomic balance with significant electrophysiological and clinical implications. Left SG stimulation causes complex ventricular repolarization changes, initially prolonging and then shortening repolarization, particularly in the lateral and posterior left ventricle, which increases dispersion and promotes arrhythmias during ischemia or long-QT syndrome [173,174,175]. In humans, percutaneous SG stimulation is feasible during electrophysiology procedures, provoking blood pressure changes and arrhythmia patterns without severe complications [176,177]. Low-level ultrasound- and fluoroscopy-guided stimulation produces inotropic but not chronotropic responses, accompanied by increased repolarization heterogeneity [178].

Autonomic effects include increased sympathetic outflow, which raises heart rate and blood pressure, as well as altered pulmonary vascular impedance [179]. Noninvasive electromagnetic SG stimulation reduces neural activity and ventricular arrhythmia incidence post-myocardial infarction in animal models, suggesting therapeutic potential [180]. SG stimulation also decreases cochlear vessel conductivity through fibres that do not pass through the superior cervical ganglia [21].

Chronic or subthreshold electrical SG stimulation induces nerve sprouting and sympathetic hyperinnervation, increasing the risk of sudden cardiac death [181]. Conversely, interventions that suppress SG activity, including vagus nerve stimulation or subcutaneous nerve stimulation, reduce arrhythmia burden and promote protective neural remodelling [182,183,184]. These findings establish SG stimulation as both a research tool for understanding autonomic cardiac control and a potential therapeutic target for arrhythmia management through modulation of sympathetic tone.

Radiofrequency ablation (RFA) of the SG provides longer-lasting sympathetic blockade compared to local anaesthetic blocks. Pulsed radiofrequency extends the effects of SGB beyond the typical few hours, proving effective for various chronic, refractory pain syndromes. It can be performed safely under ultrasound guidance for complex regional pain syndromes [7,28]. Continuous RFA, typically using temperatures below 75 °C to preserve motor nerve fibres, offers a minimally invasive alternative to surgical denervation using CT or ultrasound guidance [7].

Bilateral SG RFA demonstrates promising results for electrical storm, with patients remaining free of recurrent ventricular arrhythmias at 22-month follow-up without procedural complications, providing a feasible alternative to surgical denervation in unstable patients [185]. Both continuous and pulsed RFA effectively treat chronic pain conditions, including complex regional pain syndrome, postherpetic neuralgia, and post-mastectomy pain, with longer-lasting relief than single-shot blocks [7,150,153,154,186].

The safety profile shows low and insignificant complication rates, with most adverse effects being minor and transient, including temporary Horner’s syndrome or mild neuralgia [7,185]. Thermal RFA may provide greater sustained relief than pulsed RFA in some chronic pain syndromes [153], though both modalities demonstrate superiority over repeated local anaesthetic blocks [7,150,153].

SGB significantly impacts conditions associated with immune dysfunction, with reports spanning a century, although the mechanisms remained poorly understood until recently [17]. SGB effectively treats immune-mediated conditions, including allergic diseases, asthma, atopic dermatitis, and ulcerative colitis, through modulation of neuro-immunomodulatory reflexes that start at the SG and end in postganglionic sympathetic fibres throughout the body [17].

The SG influences immune function through dual mechanisms. The direct pathway involves SG neural branches innervating the thymus gland, modulating thymic activity and T-lymphocyte production/maturation [17]. The indirect path operates through afferent nerve pathways projecting to key brain regions, including the amygdala, insula, and hippocampus, which maintain bidirectional connections with the locus coeruleus (LC), the primary noradrenergic control centre [17,187]. The LC regulates autonomic nuclei that send sympathetic efferent fibres to both primary lymphoid organs (bone marrow, thymus) and secondary lymphoid tissues (spleen, lymph nodes, mucosa-associated lymphoid tissue) [17,187]. The anatomical circuit from LC to SG involves descending noradrenergic projections through the medulla and pons, synapses on preganglionic sympathetic neurons in the intermediolateral cell column at T1-T4, and preganglionic fibres travelling to synapse with postganglionic neurons in the SG [187]. This dual mechanism suggests that the SG blockade influences immune function through both direct thymic innervation and complex central nervous system pathways affecting multiple components of the immune system [17].

Thoracic outlet syndrome (TOS) involves the compression of neurovascular structures passing through the superior thoracic outlet, with the first rib serving as a common denominator [188]. While typically classified as neurogenic, venous, or arterial based on the compressed structure, the SG’s proximity to the first rib allows for compression, potentially contributing to sympathetic symptoms [189,190,191]. Patients may present with pain syndromes, hyperhidrosis, or vasomotor changes, some mediated by SG involvement, making the SGB useful diagnostically and therapeutically [192]. Congenital anomalies (cervical ribs), trauma, or muscle hypertrophy increase compression risk [190,191]. Sympathetic hyperactivity from SG compression against the first rib may produce Raynaud’s syndrome, requiring transaxillary first rib resection with dorsal sympathectomy [188]. Despite the anatomical plausibility, most TOS literature focuses on neurovascular compression, with limited direct evidence of SG compression as a primary mechanism.

Stellectomy, the surgical removal of the SG, has been extensively studied for cardiac arrhythmia management and demonstrates distinct laterality-dependent effects. Right stellectomy significantly reduces heart rate and impairs high-workload exercise capacity, while left stellectomy increases heart rate and coronary flow without impairing contractility due to right SG compensation [193]. These procedures have opposite effects on ventricular refractory periods: right stellectomy decreases refractoriness, while left stellectomy increases it, with bilateral stellectomy showing increased refractoriness primarily due to the left-sided contribution [194]. Left stellectomy demonstrates significant antiarrhythmic properties by raising the ventricular fibrillation threshold and reducing arrhythmia incidence, particularly after myocardial infarction or in long-QT syndrome [195,196,197,198]. The antiarrhythmic mechanism involves reduced sympathetic drive, increased cardiac electrical stability, and anti-inflammatory signalling through JAK2-STAT3 pathway activation [197,199]. Unlike denervation in other contexts, left stellectomy does not cause supersensitivity to norepinephrine; rather, it reduces arrhythmogenic responses [200]. Left stellectomy also enhances coronary bed dilation and improves endocardial perfusion, indicating tonic sympathetic influence on coronary blood flow [201]. Bilateral thoracoscopic stellectomy (cardiac sympathetic denervation, CSD) provides significant antiarrhythmic effects in patients with refractory ventricular arrhythmias unresponsive to medications and ablation [149,202]. Clinical studies report reduction or cessation of ventricular tachyarrhythmias and implantable cardioverter-defibrillator shocks without causing hemodynamic compromise [203,204]. The procedure is typically performed via video-assisted thoracoscopic surgery, less invasive than open surgery but carrying specific risks [203,204]. Perioperative complications include cardiogenic shock, vasoplegia, and the need for inotropic or mechanical support in high-risk patients, with some requiring ICU admission and prolonged hospitalization [202]. However, major surgical complications, including Horner’s syndrome, remain rare, with most studies reporting low overall complication rates and acceptable long-term survival in patients with otherwise limited options [202,203,204]. Off-target effects include lung injury, compensatory hyperhidrosis, and Horner’s syndrome [205]. Minimally invasive alternatives, such as transtracheal cardiac plexus block, are being explored to achieve similar antiarrhythmic effects with reduced morbidity, potentially benefiting patients unsuitable for surgery [205]. Additionally, bilateral stellectomy significantly decreases cerebrovascular resistance, with the most significant impact in patients with initially slow cerebral blood flow and marked increases in cerebrovascular resistance [206].

In neurodegenerative disorders, the SG shows signs of inflammation, oxidative stress, and neurochemical remodelling, which may contribute to autonomic and neurological symptoms. Research on human SG from patients with advanced heart disease and arrhythmias, a population with overlapping neurodegenerative and autonomic dysfunction, reveals several key changes: increased inflammation, oxidative stress, neurochemical remodelling, and glial activation [207]. There is greater infiltration of immune cells (T cells, neutrophils) and activation of SGCs in the SG compared to controls. Neurons in the SG show more lipofuscin deposits (a marker of oxidative damage) and mitochondrial degeneration [207]. There is a reduction in adrenergic (sympathetic) markers and altered neurotransmitter profiles, indicating disrupted sympathetic signalling. The SGCs surrounding SG neurons are more activated, which may amplify or dysregulate sympathetic output [207]. These changes are thought to contribute to excessive or dysfunctional sympathetic tone, which can worsen arrhythmias and other autonomic symptoms seen in neurodegenerative and systemic diseases [207]. Case reports and animal studies suggest that SG dysfunction or targeted interventions (like SGB) may influence symptoms such as pain, cognitive decline, and autonomic instability in neurodegenerative conditions, including corticobasal syndrome, fatal familial insomnia, and postoperative cognitive dysfunction [147,208,209]. The SG’s role in modulating inflammation and neuroimmune signalling may be relevant across a range of neurodegenerative disorders [17,147,207].

## 7. Knowledge Gaps and Future Research Directions

Despite significant advances in understanding the SG anatomy and the expanding clinical applications of SGB, several significant knowledge gaps remain regarding anatomical research, clinical evidence, mechanistic understanding, and technological advances (Table 8).

Future research should prioritize prospective multicenter studies with standardized methodologies to establish evidence-based guidelines for SGB applications across various clinical scenarios. Integration of advanced imaging with clinical outcomes will be crucial for personalized approaches to sympathetic interventions.

## 8. Conclusions

The SG represents a critical anatomical structure whose clinical significance extends far beyond its traditional role in sympathetic innervation. This comprehensive review reveals the complexity of SG anatomy, the expanding therapeutic applications of SGB, and the evolving understanding of its physiological mechanisms. Significantly, this work demonstrates the feasibility and clinical utility of high-resolution MRI for non-invasive SG visualization—a novel contribution to autonomic nervous system imaging that bridges the gap between cadaveric anatomical studies and clinical practice.

Our original MRI imaging demonstrates that the SG can be consistently identified at the thoracic inlet with its characteristic relationships to surrounding structures: anterior to the neck of the first rib, lateral to the longus colli muscle, and posterior to the vertebral artery. This imaging-based anatomical localization, previously underutilized in clinical practice, provides a foundation for personalized procedural planning and may explain variable outcomes in SGB procedures. The integration of high-resolution imaging with detailed anatomical knowledge enables clinicians to predict and navigate anatomical variations, including aberrant vascular anatomy and variant ganglion positions, thereby improving procedural safety and efficacy.

The SG stands at the intersection of anatomy, physiology, and clinical therapeutics. Its complex anatomical relationships, sophisticated cellular organization, and expanding therapeutic applications reflect the evolving understanding of autonomic nervous system function. As diagnostic and therapeutic technologies continue to advance, particularly in the MRI and image-guided interventions, the SG will likely assume an increasingly important role in personalized medicine approaches to autonomic dysfunction.

The translation of anatomical knowledge into clinical practice requires ongoing collaboration between anatomists, clinicians, and researchers. This interdisciplinary approach, enhanced by advanced imaging capabilities demonstrated in this work, will be essential for optimizing patient outcomes while advancing our understanding of this remarkable neural structure. The SG exemplifies how detailed anatomical knowledge, when integrated with modern imaging technology, clinical innovation, and technological advancement, can transform therapeutic possibilities and improve patient care across multiple medical specialties. Future research should focus on expanding the application of MRI for routine SG assessment and correlating imaging-defined anatomical variants with clinical outcomes in SGB procedures.

## Figures and Tables

**Table 8:** The future research targets regarding the stellate ganglion (SG) and SG block (SGB). SGCs: satellite glial cells.

Knowledge Gaps	Future Research Directions
Anatomical research needs	- Systematic large-scale cadaveric studies quantifying the prevalence of anatomical variants across different populations; - Three-dimensional reconstruction studies using high-resolution imaging to create comprehensive anatomical atlases; - Correlation studies between anatomical variants and clinical outcomes of SGB; - Developmental studies examining the embryological basis for SG variability.
Clinical evidence	- Large-scale randomized controlled trials for most SGB indications beyond pain management; - Standardized protocols for SGB technique, including optimal injection volumes, anaesthetic concentrations, and anatomical approaches; - Long-term outcome studies for SGB in cardiac arrhythmias, PTSD, and immune dysfunction; - Comparative effectiveness studies between different SGB techniques (fluoroscopy vs. ultrasound guidance); - Systematic evaluation of SGB complications with prospective registries.
Mechanistic understanding	- The precise pathways through which SGB affects the immune function and neuroimmune communication; - The role of intermediate ganglia and nerve of Kuntz in incomplete SGB response; - Relationship between SGCs activation and sympathetic dysfunction in disease states; - The central neuroplastic changes following peripheral sympathetic modulation.
Technological advances	- Development of MRI neurography protocols for routine SG visualization; - Advanced ultrasound techniques for real-time identification of anatomical variants; - Novel neuromodulation approaches (radiofrequency, cryoablation, focused ultrasound) requiring anatomical validation; - Artificial intelligence algorithms for predicting SGB success based on patient-specific anatomy.

## Data Availability

The raw data supporting the conclusions of this article will be made available by the authors on request.

## Supplementary Materials

The following supporting information can be downloaded at: https://www.mdpi.com/article/10.3390/diagnostics15222911/s1. Figure S1: The left sympathetic cervical trunk; Figure S2: Region of the right inferior cervical ganglion; Figure S3. Transmission electron microscopy of the rat stellate ganglion.

## Abbreviations

The following abbreviations are used in this manuscript:

AS	Ansa Subclavia (subclavian ansa of Vieussens)
BDNF	Brain-Derived Neurotrophic Factor
CBT-I	Cognitive Behavioral Therapy for Insomnia
CRPS	Complex Regional Pain Syndrome
CSD	Cardiac Sympathetic Denervation
CT	Computed Tomography
Cx43	Connexin 43
EAAT1/EAAT2	Excitatory Amino Acid Transporter 1/2
EMDR	Eye Movement Desensitization and Reprocessing
GAD	Generalized Anxiety Disorder
GDNF	Glial Cell Line-Derived Neurotrophic Factor
GFAP	Glial Fibrillary Acidic Protein
GLAST	Glutamate Aspartate Transporter
GLT1	Glutamate Transporter 1
GRP	Gastrin-Releasing Peptide
HBO	Hyperbaric Oxygen
HRT	Hormone Replacement Therapy
ICCN	Inferior Cervical Cardiac Nerve
ICD	Implantable Cardioverter Defibrillator
IG	Intermediate Ganglion
IN	Inferior Cervical Cardiac Nerve
INR	International Normalized Ratio
JAK2STAT3	Janus Kinase 2 Signal Transducer and Activator of Transcription 3
LA	Local Anesthetic
LC	Locus Coeruleus
LHA	Lateral Hypothalamic Area
MCA	Middle Cerebral Artery
MG	Middle Cervical Ganglion
MN	Middle Cardiac Nerve
MRI	Magnetic Resonance Imaging
NGF	Nerve Growth Factor
Orai1	Calcium Release Activated Calcium Modulator 1
PSQI	Pittsburgh Sleep Quality Index
PTSD	Post-Traumatic Stress Disorder
RFA	Radiofrequency Ablation
RLN	Recurrent Laryngeal Nerve
SAH	Subarachnoid Hemorrhage
SG	Stellate Ganglion
SGB	Stellate Ganglion Block
SGCs	Satellite Glial Cells
SIF	Small Intensely Fluorescent (cells)
SN	Superior Cardiac Nerve
SOCE	Store-Operated Calcium Entry
SR	Systematic Review
SSRIs	Selective Serotonin Reuptake Inhibitors
STIM1	Stromal Interaction Molecule 1
TENS	Transcutaneous Electrical Nerve Stimulation
TN	Thoracic Cardiac Nerve
TOS	Thoracic Outlet Syndrome
US	Ultrasound
VN	Vertebral Nerve
VT/VF	Ventricular Tachycardia/Fibrillation
