# Supplementary figures and images for: Anatomy, Imaging, and Clinical Significance of the Cervicothoracic (Stellate) Ganglion
Source: Diagnostics (Basel). 2025 Nov 17;15(22):2911. doi: 10.3390/diagnostics15222911 (PMC12651590; doi:10.3390/diagnostics15222911)

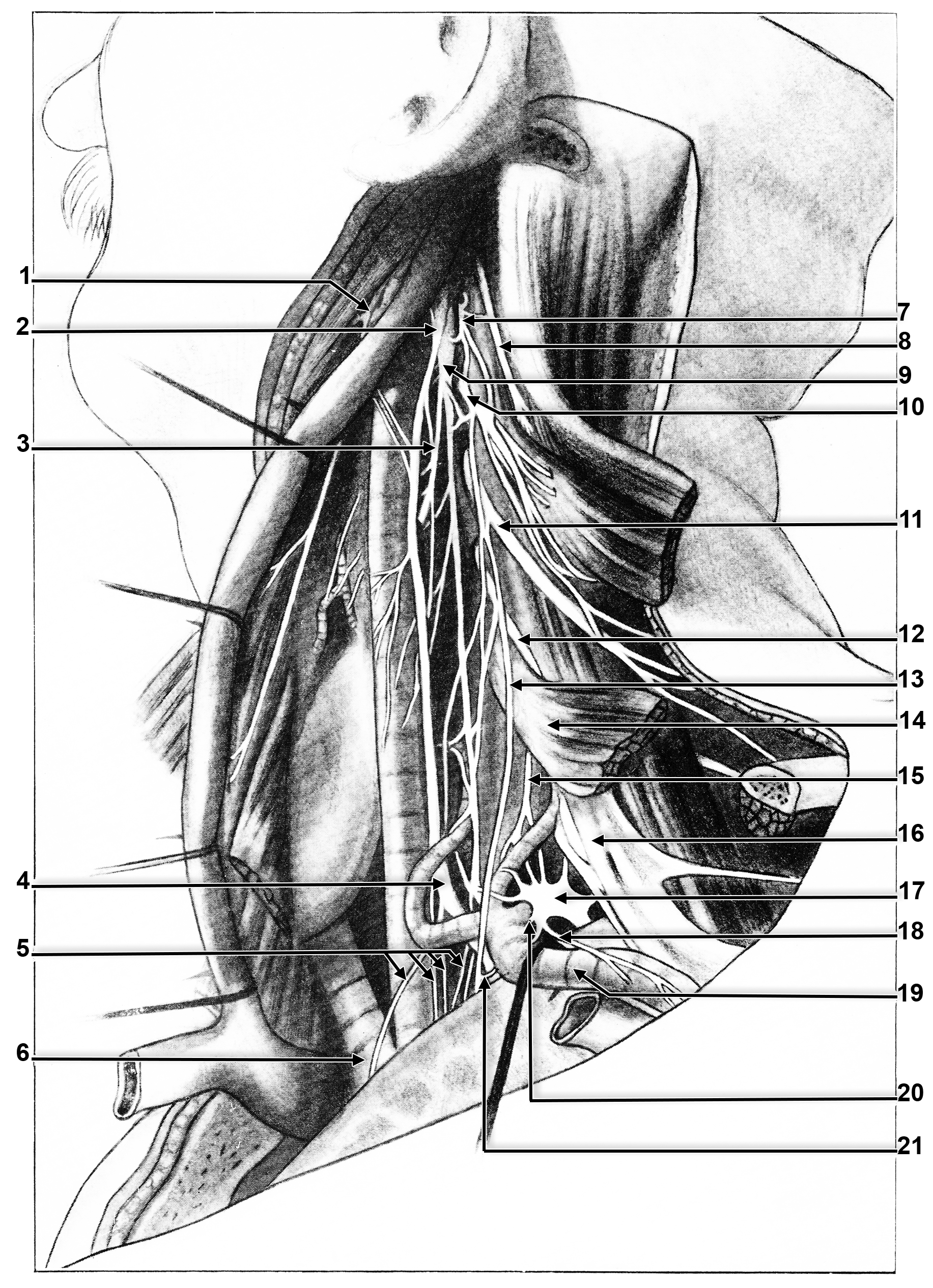

Supplement: Supplementary file 1 [file diagnostics-15-02911-s001.zip › figure S1.tif]

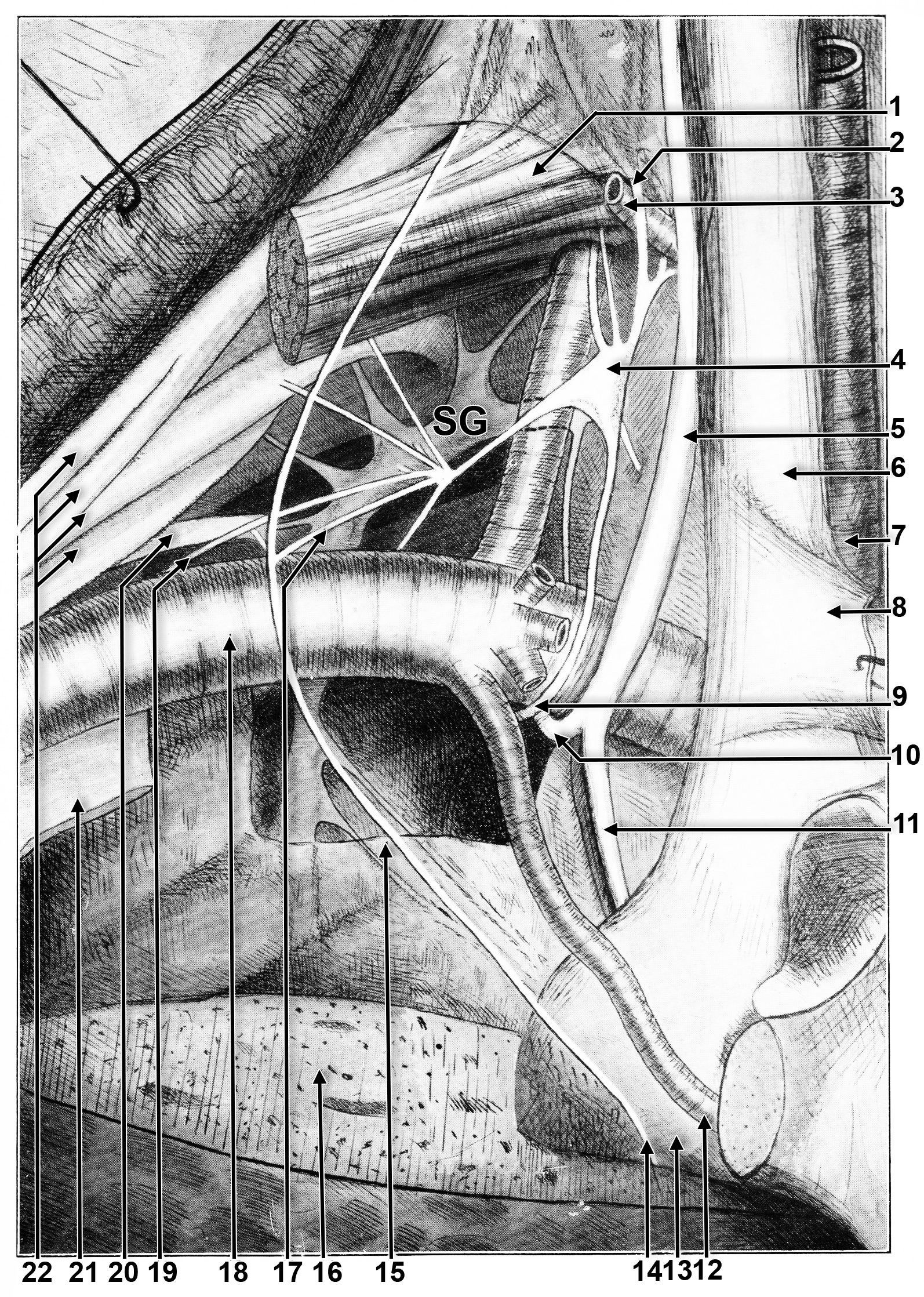

Supplement: Supplementary file 1 [file diagnostics-15-02911-s001.zip › FIgure S2.tif]

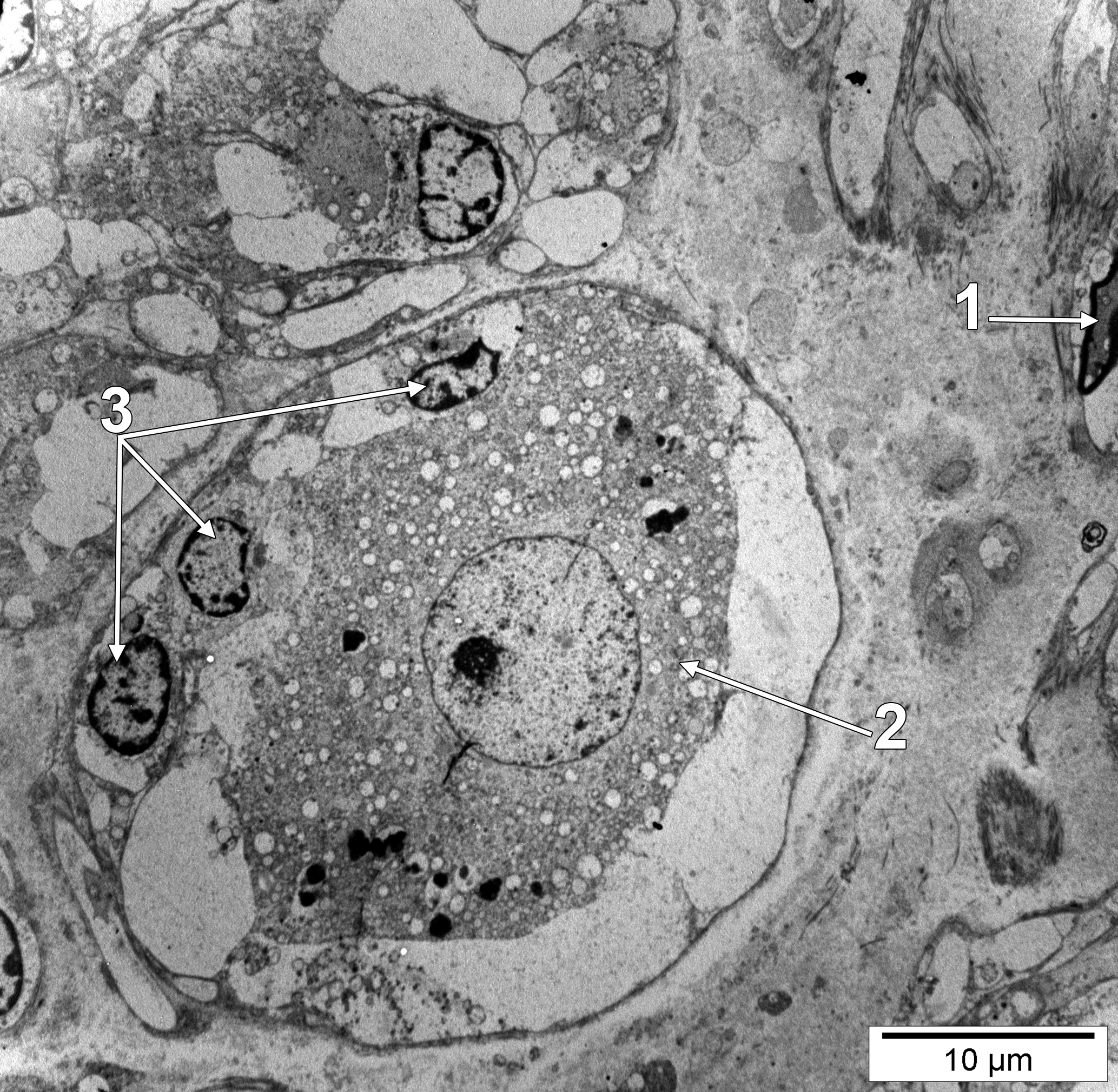

Supplement: Supplementary file 1 [file diagnostics-15-02911-s001.zip › Figure S3.tif]
